# Analysis of single nuclear chromatin accessibility reveals unique myeloid populations in human pancreatic ductal adenocarcinoma

**DOI:** 10.1002/ctm2.1595

**Published:** 2024-03-01

**Authors:** Hillary G. Pratt, Li Ma, Sebastian A. Dziadowicz, Sascha Ott, Thomas Whalley, Barbara Szomolay, Timothy D. Eubank, Gangqing Hu, Brian A. Boone

**Affiliations:** ^1^ Cancer Cell Biology West Virginia University Morgantown West Virginia USA; ^2^ WVU Cancer Institute West Virginia University Morgantown West Virginia USA; ^3^ Department of Microbiology Immunology and Cell Biology West Virginia University Morgantown West Virginia USA; ^4^ Warwick Medical School University of Warwick Coventry UK; ^5^ School of Biosciences Cardiff University Cardiff UK; ^6^ Division of Infection and Immunity & Systems Immunity Research Institute Cardiff University Cardiff UK; ^7^ In Vivo Multifunctional Magnetic Resonance Center West Virginia University Morgantown West Virginia USA; ^8^ Department of Surgery West Virginia University Morgantown West Virginia USA

**Keywords:** ATAC‐Seq, myeloid, PDAC

## Abstract

**Background:**

A better understanding of the pancreatic ductal adenocarcinoma (PDAC) immune microenvironment is critical to developing new treatments and improving outcomes. Myeloid cells are of particular importance for PDAC progression; however, the presence of heterogenous subsets with different ontogeny and impact, along with some fluidity between them, (infiltrating monocytes vs. tissue‐resident macrophages; M1 vs. M2) makes characterisation of myeloid populations challenging. Recent advances in single cell sequencing technology provide tools for characterisation of immune cell infiltrates, and open chromatin provides source and function data for myeloid cells to assist in more comprehensive characterisation. Thus, we explore single nuclear assay for transposase accessible chromatin (ATAC) sequencing (snATAC‐Seq), a method to analyse open gene promoters and transcription factor binding, as an important means for discerning the myeloid composition in human PDAC tumours.

**Methods:**

Frozen pancreatic tissues (benign or PDAC) were prepared for snATAC‐Seq using 10× Chromium technology. Signac was used for preliminary analysis, clustering and differentially accessible chromatin region identification. The genes annotated in promoter regions were used for Gene Ontology (GO) enrichment and cell type annotation. Gene signatures were used for survival analysis with The Cancer Genome Atlas (TCGA)‐pancreatic adenocarcinoma (PAAD) dataset.

**Results:**

Myeloid cell transcription factor activities were higher in tumour than benign pancreatic samples, enabling us to further stratify tumour myeloid populations. Subcluster analysis revealed eight distinct myeloid populations. GO enrichment demonstrated unique functions for myeloid populations, including interleukin‐1b signalling (recruited monocytes) and intracellular protein transport (dendritic cells). The identified gene signature for dendritic cells influenced survival (hazard ratio = .63, *p* = .03) in the TCGA‐PAAD dataset, which was unique to PDAC.

**Conclusions:**

These data suggest snATAC‐Seq as a method for analysis of frozen human pancreatic tissues to distinguish myeloid populations. An improved understanding of myeloid cell heterogeneity and function is important for developing new treatment targets in PDAC.

## Graphical abstract

1. snATAC‐Seq can be used to assay frozen benign pancreatic and PDAC tissues.

2. snATAC‐Seq data can be used to determine the regulome and molecular characteristics of cell populations.

3. Myeloid cells from the PDAC TME can be characterized using snATAC‐Seq.

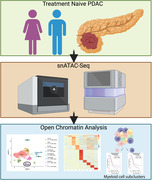

## 1 INTRODUCTION

Although significantly improved over the past decade, the 5‐year survival rate for pancreatic ductal adenocarcinoma (PDAC) is only 12%. The unique stromal makeup of the PDAC tumour, including activated fibroblasts and suppressive immune cells, is a critical component driving tumour progression and resistance to therapy. To enhance therapeutic options for PDAC patients, a better understanding of this unique tumour microenvironment (TME) is needed.

Immune cell infiltration of the PDAC TME is an important subject of inquiry as immunotherapies with success in other cancer types have failed in PDAC.^1^ In particular, the myeloid component of the PDAC TME has been identified as having a critical role in this immunosuppression and treatment failure. Circulating monocytes were correlated with a worse overall survival in PDAC patients^2^; however, clinical trials blocking monocyte recruitment into the tumour combined with FOLFIRINOX^3^ or gemcitabine/nab‐paclitaxel^4^ demonstrated limited efficacy. Another population of myeloid cells, tissue‐resident macrophages (TRMs), are present before tumour initiation and are the prominent source of immunosuppressive, pro‐fibrotic macrophages in the PDAC TME in murine models.^5^ Meanwhile, M1 and M2 remain the most common characterisation of macrophage populations in the TME, but individual macrophages exist along more of a continuum than this nomenclature reflects.^6^ Such studies demonstrate a need for refined characterisation of myeloid source and function in the PDAC TME to understand pathogenesis and uncover novel therapeutic strategies.

Recently, single cell sequencing technologies have been harnessed to determine cell function and composition in the TME. Single cell RNA sequencing (scRNA‐Seq) has demonstrated heterogeneity between patients in the tumour and stromal components of PDAC.^7^, ^8^, ^9^, ^10^, ^11^, ^12^ Since stroma can comprise much of the tumour volume,^8^, ^13^ scRNA‐Seq has been employed to identify the immune cell milieu and ascertain how these cells influence tumour progression,^10^ patient prognosis^7^, ^11^, ^12^ and chemotherapy response.^11^ Given the importance of myeloid cells in PDAC progression,^2^, ^5^ scRNA‐Seq studies have also explored the phenotype and function^11^, ^12^ as well as source,^12^ of macrophages. Although the transcriptome of cells within the PDAC TME has been heavily studied,^11^, ^14^, ^15^, ^16^ there has been limited progress on the regulome of cells within the TME. To answer such questions, a method for analysing open chromatin, termed assay for transposase accessible chromatin sequencing (ATAC‐Seq), can explore the epigenetic underpinnings of diseases.^17^, ^18^ Open chromatin can also be utilised for cell identification due to its role in regulation of gene expression.^18^, ^19^ A limited number of studies exploring single cell chromatin accessibility from freshly isolated PDAC specimens have shown an important role for KRAS regulation of enhancer activity^20^ and dysregulation of methylation^7^ in PDAC progression, demonstrating the importance of epigenomics in understanding pancreatic diseases. Furthermore, open chromatin and transcription factor activity can be used to determine myeloid cell origin,^19^ which is relevant for understanding the highly plastic myeloid populations that are present in the PDAC tumour and the role for these cells in cancer progression.

In the present study, we applied single nuclear ATAC‐Seq (snATAC‐Seq) to frozen benign and PDAC patient pancreatic specimens. Our results justified snATAC‐Seq as an effective method for retrospective analysis of tumours to identify unique cell populations within the TME. Through snATAC‐Seq, we combined information on transcription factor binding and gene promoter activity to identify cell populations and infer their functions in benign pancreatic and PDAC samples. Utilising these findings, we then explored the impact of distinct myeloid cell populations on patient survival.

## 2 MATERIALS AND METHODS

### 2.1 Patient tissue collection

Prior to sample collection, Institutional Review Board approval was obtained from West Virginia University (#1903496995) and all patients signed informed consent. All patients underwent surgical resection with pancreaticoduodenectomy as part of standard of care. Benign and PDAC samples for snATAC‐Seq were treatment naïve. Tissue was obtained from the pathology frozen section suite immediately upon surgical resection. Diagnosis was confirmed by pathology. PDAC tumour tissue or benign pancreatic tissue was frozen alone (nucleus isolation) or in OCT (optimal cutting temperature compound; immunofluorescence staining) and stored at −80°C.

### 2.2 Immunofluorescent staining

Sections (10 μm) of OCT‐embedded frozen tissues were fixed in 10% formalin then permeabilised with .1% Triton‐X for 10 min and blocked for 1 h at room temperature with 5% bovine serum albumin (BSA; MACS BSA stock solution, Miltenyi Biotec, 130‐091‐376) and 10% goat serum (Gibco, 16210‐072) in Dulecco's Phosphate Buffered Saline (DPBS;Gibco, 14190144). Slides were incubated with primary antibodies (1:100) overnight at 4°C (mouse anti‐human CD11c [Invitrogen, 14‐0116‐82], mouse anti‐human CD11b [Invitrogen, 14‐0118‐82], rabbit polyclonal SEMA4A [Invitrogen, PA5‐101258] and rabbit anti‐human TRIM29 [Invitrogen, 703612]). Tissues were incubated with secondary antibody for 1 h at room temperature (Alexa Fluor 488 goat anti‐rabbit [Invitrogen, A‐11008]; Alexa Fluor 647 goat anti‐mouse [Invitrogen, A‐21235]). Vector TrueVIEW Autofluorescence Quenching Kit (Vector Laboratories, SP‐8400‐15) was prepared per manufacturer's instructions and added for 5 min. Then, the slide was stained with DAPI (Thermo Scientific, 62248). Vectashield Vibrance Antifade Mounting Media (Vector Laboratories, H‐1700) was used to mount coverslips. All antibodies were diluted in 1% BSA and 10% goat serum in DPBS. Slides were washed with DPBS between steps. Imaging was performed on a Nikon A1R confocal microscope using Nikon A1R software with 60× zoom with oil immersion.

### 2.3 Nuclei isolation from frozen tissue

Isolation procedure was adapted from nuclei isolation from complex tissues for Single Cell Multiome ATAC + Gene Expression Sequencing (CG000375). Nuclei were isolated from 50 mg of frozen tissue cut into five small sections with a razor blade. The tissue pieces were homogenised for 1.5 min with a tissue homogeniser and pestle in 10 mM Tris–HCl, 10 mM NaCl, 3 mM MgCl_2_, .1% Nonidet P40 substitute and 1 mM dithiothreitol (DTT) in nuclease‐free water. Tissue was dissociated in lysis buffer for 5 min before passing through a 70 μm cell strainer. The solution was then centrifuged (500 × *g*, 4°C, 5 min). The supernatant was removed and replaced with 1 mL 1% BSA in DPBS and allowed to incubate for 5 min before resuspension. The nuclei were centrifuged again (500 × *g*, 4°C, 5 min). The pellet was resuspended in 1 mL 1% BSA in DPBS and passed through a 30 μm cell strainer. All steps were performed on ice. Nuclei were stained with 7‐aminoactinomycin D (7‐AAD) readymade solution 1:100 (Sigma, SML‐1633) and sorted with a FACSAria III Cell Sorter. Nuclei were collected into 10% BSA. For snMultiomics, 1 U/mL RNase inhibitor (Sigma–Aldrich, 03335402001) was added to all final solutions.

### 2.4 Bulk ATAC‐Seq

The Omni‐ATAC Protocol^18^ was followed. Libraries were prepared with sorted nuclei with a Tagment DNA Enzyme and Buffer Large Kit (Illumina, 20034198) and run on 2% agarose gel to observe the nucleosome phasing pattern and size select. The gel was cut for an approximately ∼200−600 bp band and subjected to gel elution using the Qiagen gel MinElute Gel Extraction Kit (Qiagen, 28606).

### 2.5 snATAC‐Seq library preparation

Libraries for snATAC‐Seq were prepared as recommended by 10× Genomics using a Chromium Single Cell ATAC Library & Gel Bead Kit (10× Genomics, 1000176). Briefly, sorted nuclei were centrifuged (500 × *g*, 4°C, 5 min). The pellet was resuspended in 100 mL .1× lysis buffer (10 mM Tris–HCl, 10 mM NaCl, 3 mM MgCl_2_, 1% BSA, 1 mM DTT, .01% Tween‐20, .01% Nonidet P40 substitute and .001% digitonin in nuclease‐free water) and incubated on ice for 2 min. Nuclei were washed with 1 mL wash buffer (10 mM Tris–HCl, 10 mM NaCl, 3 mM MgCl_2_, 1% BSA, .1% Tween‐20 and 1 mM DTT in nuclease‐free water) and centrifuged (500 × *g*, 4°C, 5 min). The pellet was resuspended in 1× nuclei buffer diluted in nuclease‐free water. The ATAC reaction was carried out as recommended by 10× Genomics. Nuclei were loaded onto the 10× Chromium Chip E (10× Genomics, 2000171) on the chromium controller to obtain 10 000 nuclei. Library quality was confirmed with an Agilent Bioanalyser. Libraries were sequenced by an Illumina NextSeq 2000 sequencer at Marshall University Genomics Core Facility.

### 2.6 snMultiome library preparation

Libraries for snMultiome + ATAC were prepared as recommended by 10× Genomics using a Chromium Single Cell Multiome + ATAC Library and Gel Bead Kit (10× Genomics, 1000283). Briefly, sorted nuclei were centrifuged (500 × *g*, 4°C, 5 min). The pellet was resuspended in 100 mL .1× lysis buffer (10 mM Tris–HCl, 10 mM NaCl, 3 mM MgCl_2_, 1% BSA, 1 U/mL RNase inhibitor, 1 mM DTT, .01% Tween‐20, .01% Nonidet P40 substitute and .001% digitonin in nuclease‐free water) and incubated on ice for 2 min. Nuclei were washed with 1 mL wash buffer (10 mM Tris–HCl, 10 mM NaCl, 3 mM MgCl_2_, 1% BSA, 1 U/mL RNase inhibitor, .1% Tween‐20 and 1 mM DTT in nuclease‐free water) and centrifuged (500 × *g*, 4°C, 5 min). The pellet was resuspended in 1× nuclei buffer with 1 U/mL RNase inhibitor and 1 mM DTT in nuclease‐free water. The ATAC and cDNA reactions were carried out as recommended by 10× Genomics. Nuclei were loaded onto the 10× Chromium Chip J (10× Genomics, 1000230) on the chromium controller for 10 000 nuclei. Library quality was confirmed with an Agilent Bioanalyser. Libraries were sequenced by an Illumina NextSeq 2000 sequencer at Marshall University Genomic Core Facility.

### 2.7 snATAC‐Seq data pre‐processing and clustering analysis

snATAC‐Seq reads were aligned to the GRCH38(hg38) reference genome. Fragment counts were quantified using cellranger‐atac count (version 2.1.0). Seurat^21^ (version 4.2.1) and Signac^22^ (version 1.7.0) were used for downstream analysis. Quality control was based on library features with settings: 1000 < nCount_ATAC (number of fragments) < 20 000, 500 < nFeature_ATAC (number of peaks) < 10 000, blacklist_fraction < 5%, nucleosome_signal < 2, TSS.enrichment > 2 and pct_reads_in_peaks (fraction of fragments within ATAC‐Seq peaks) > 15%. Nucleosome_signal (strength of the nucleosome signal), TSS.enrichment (transcription start site [TSS] enrichment score as defined by ENCODE) and blacklist_fraction (ratio reads in genomic blacklist regions as defined by ENCODE) were calculated by the function NucleosomeSignal, TSSEnrichment and FractionCountsInRegion of Signac, respectively. RunTFIDF was used to compute term‐frequency inverse‐document‐frequency normalisation on the matrix. FindTopFeatures was used to find the most frequently observed features. RunSVD was used to find the top 50 largest singular value and corresponding singular vectors of the matrix. RunHarmony^23^ was used to correct batch effects. RunUMAP was used for dimensional reduction and visualisation via uniform manifold approximation and projection (UMAP). FindNeighbours was used to compute the nearest neighbours for the object. FindClusters was used to identify clusters of cells by a shared nearest neighbours modularity optimisation based on Smart Local Moving (SLM) clustering algorithms^24^ with a resolution of .2. FindAllMarkers was used to identify the differentially accessible regions for each cluster by against all other clusters, by setting ‘LR’ as the test used, peak_region_fragments as the test variable, and logfc.threshold (fold change in log2 scale) as .25.

### 2.8 Cell type annotation for the snMultiome dataset

Sequencing reads were aligned to the GRCH38 (hg38) reference genome and quantified using cellranger‐arc count (version 2.0.2). Quality control was based on each library's features with these settings: 500 < nCount_ATAC < 40 000, 500 < nCount_RNA (total number of RNA fragments) < 20 000, 100 < nFeature_ATAC < 20 000, 100 < nFeature_RNA (number of genes) < 5000, nucleosome_signal < 2, pct_reads_in_peaks > 15%, percent.mt < 2%, percent.ribo < 2%, TSS.enrichment > 2 and blacklist_fraction < 2%. Percent.mt (RNA reads from mitochondrial genes) and percent.ribo (reads from ribosomal genes) were calculated by the PercentageFeatureSet function.

The RNA part of the snMultiome was used to annotate cell type and the ATAC part for validation of the cell type annotation in the snATAC‐Seq (next section). For the RNA part, Seurat^21^ (version 4.2.1) was used for downstream analysis. NormalizeData was used for normalisation and set normalisation method as ‘CLR’ (centered log ratio transformation). Cell cycle phase score was calculated by CellCycleScoring. Variable features were identified by FindVariableFeatures and the top 2000 were selected. Data were scaled and centered by ScaleData function and regress out the cell cycle's effect. RunPCA was used for principal component analysis (PCA) dimensionality reduction. RunHarmony^23^ was used to integrate the dataset. RunUMAP and FindNeighbours were called as in the previous section. FindClusters was used to identify clusters of cells by a shared nearest neighbours modularity optimisation based on original Louvain clustering algorithms with a resolution of .2. Cell clusters annotated with marker genes are acinar cell (*CUZD1* ^25^), acinar to ductal metaplasia (ADM) cell (*ONECUT1* ^26^), ductal cell (*KRT19* ^11^, ^15^), endocrine cell (*PAX6* ^27^), endothelial cell (*TEK* ^28^), fibroblast (*COL1A2* ^29^), myeloid cell (*CD163*,^12^ *CSF2RA* ^30^) and T cell (*IL7R* ^11^) (Table S1). After annotation, an extended list of marker genes was predicted for each cell type against all other cell types through FindAllMarkers with ‘wilcox’ test and logfc.threshold as .25 (Table S1).

### 2.9 Cell type annotation and validation for snATAC‐Seq

Cell type annotation for the snATAC‐Seq datasets was done through an integrative analysis with annotated cells from the snMultiome dataset. After differential accessible regions were identified for each cluster from the snATAC‐Seq dataset (Table S2), promoters overlapping with these peaks were used to define signature genes for each cluster. Signature genes for each cluster from the snATAC‐Seq dataset were then compared to marker genes for each cell type defined from the RNA part of the snMultiome data set for an overrepresentation analysis (observed number/expected number) (Figure S5D). For each snATAC‐seq cluster, the cell type with the highest overrepresentation was assigned to that cluster.

We validated our snATAC‐Seq annotations by four methods (see Section 3). First, expression of representative genes in snATAC‐Seq clusters was examined in the snMultiome dataset to visually validate cell type annotation. Second, we identified the overrepresented molecular pathways for snATAC‐Seq clusters with Gene Ontology (GO) enrichment of signature genes. Third, we aligned the snATAC‐Seq data with the ATAC part of our snMultiome. Cell type annotation for the ATAC part of the snMultiome data was directly transferred from the RNA part of the snMultiome data. We applied the RunHarmony function in the Harmony package^23^ for this alignment, visualised data in UMAP, projected their respective cell type annotation and visually examined their positional relationship.

Lastly, four scATAC‐Seq datasets^20^ were downloaded from Gene Expression Omnibus (GSE147726) (one patient tumour and adjacent nonmalignant tissue, another tumour and a histologically normal pancreas). While cell types are listed by Li et al.,^20^ a file documenting cell annotation is absent from their repository. We therefore reanalysed their data. Briefly, we mapped the raw reads to the hg38 reference genome. Seurat^21^ (version 4.2.1) and Signac^22^ (version 1.7.0) were used. We identified differential accessible regions of the clusters and signature genes for each cluster through overlapping analysis at promoters. We applied GO enrichment to the signature genes to identify overrepresented pathways for each cluster and used this information to annotate cell types from Li et al.^20^ We then used the RunHarmony function in the Harmony package^23^ to align our snATAC‐Seq datasets with the public scATAC‐Seq dataset, visualised cells in UMAP, projected cell type annotation from its respective data set and visually examined the positional relationship.

### 2.10 Tumour sample snATAC‐Seq dataset extraction and annotation

After annotating the benign and tumour snATAC‐Seq dataset, we extracted the tumour samples. For this extracted new object, Seurat^21^ (version 4.2.1) and Signac^22^ (version 1.7.0) were used for downstream analysis. FindClusters was used to identify clusters of cells by a shared nearest neighbours modularity optimisation based on SLM clustering algorithms^24^ with a resolution of .8, and we identified 19 subclusters. Each cluster was generally dominated by one cell type after transferring annotation from the benign and tumour samples. Clusters were designated based on the predominant cell type and merged if they shared the same cell type.

To investigate the myeloid subpopulations in PDAC tumours, we extracted the myeloid population (Table S3). Seurat^21^ (version 4.2.1) and Signac^22^ (version 1.7.0) were used. Myeloid subcluster 0 had no differentially expressed promoters (*p*‐value < .01) and was identified as a possible myeloid cell transitional state. Myeloid subclusters (>50 nuclei) were annotated—myeloid subcluster 1: recruited monocytes (*ALOX5*,^31^ *NLRC4* ^32^), myeloid subcluster 2: dendritic cells (*SEMA4A*,^33^, ^34^ *SEMA7A*,^35^ *HLA‐DRB5* ^36^ *^,^* ^37^), myeloid subcluster 3: myeloid‐derived suppressor cells (MDSCs) (*S100A9*, *CSF2RA*, *IFNGR2*)^11^, ^38^ and myeloid subcluster 4: tissue resident/epithelial‐like macrophages (*KRT7*,^11^ *KRT8*,^11^ *YAP1*,^5^ *COL16A1* ^5^).

### 2.11 ChromVAR analysis of snATAC‐Seq dataset

For the snATAC‐Seq dataset, RunChromVAR^39^ function in Signac^22^ (version 1.7.0) was used to calculate the transcription factor enrichment score. Motif matrices were obtained from the JASPAR2020 library. FindAllMarkers was used to identify differentially enriched transcription factor motifs for each subcluster against all other subclusters, ‘wilcox’ was set as the test used and logfc.threshold was set at .25.

### 2.12 GO enrichment analysis

Metascape^40^ was used for GO enrichment analysis with the complete *Homo sapiens* proteome as background. We selected the top 10 hits for visualisation.

### 2.13 Survival analysis

The top 10 genes (by *p*‐value) specific to a myeloid subcluster identified from differential accessibility analysis were used for survival analysis in the Survival Genie application.^41^ The Survival Genie Analysis Type selection criteria were ‘Gene‐Based’, ‘GENE SETS’ and ‘Upload my own’. Additional settings were select program: ‘TCGA’, select dataset: ‘TCGA‐PAAD, TCGA‐CHOL, TCGA‐COAD or TCGA‐STAD’, select tumour: ‘Primary’, select groups: ‘Median’, select survival: ‘Overall Survival’, select tumor infiltrating lymphocytes (TILS): LM22. The survival curves, hazard ratios and *p*‐values are reported.

## 3 RESULTS

### 3.1 Single nuclear analysis of open chromatin shows unique cell populations in benign pancreas and PDAC

**FIGURE 1:**
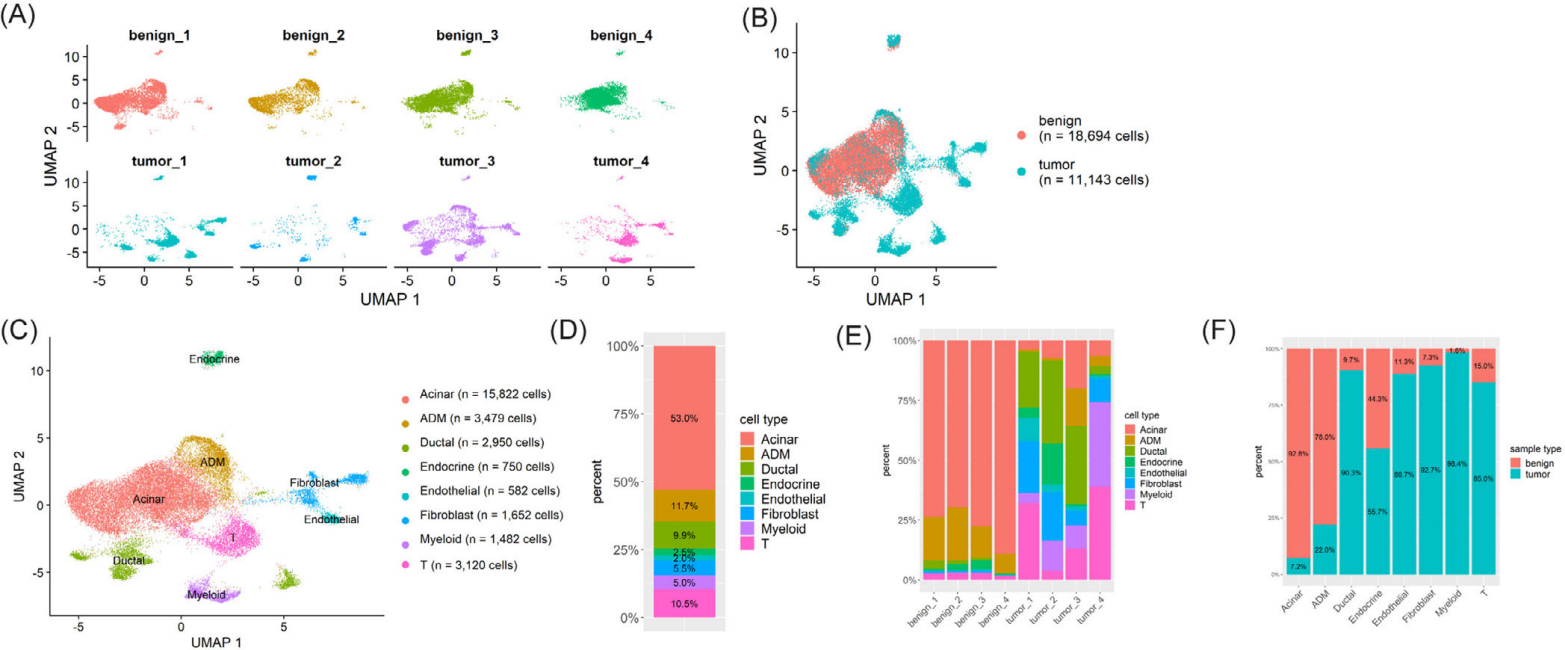
Cellular heterogeneity and unique cell population to pancreatic adenocarcinoma (PDAC). (A) Uniform manifold approximation and projection (UMAP) visualisation of benign and PDAC single nuclear assay for transposase accessible chromatin (ATAC) sequencing (snATAC‐Seq) dataset. (B) UMAP visualisation comparing benign and PDAC. (C) UMAP visualisation of the cell clusters and their annotations. (D) Cell type distribution among benign and PDAC snATAC‐Seq datasets. (E) Cell type distribution among each dataset of benign and PDAC samples, highlighting myeloid (purple) as a unique cell population presented in PDAC samples. (F) Cell distribution comparing benign and tumour contribution to cell types.

Benign pancreatic (*n* = 4) and PDAC (*n* = 4) samples were obtained following pancreaticoduodenectomy for eight total patients. Patients had pathology in the pancreatic head, which represents the majority of PDAC cases,^42^ and had no neoadjuvant chemotherapy, as neoadjuvant therapy may modulate myeloid cell populations.^2^, ^11^, ^43^ Patients were of similar sex and age, and pathology was confirmed by histopathology of the resected specimen listed in Figures S1 and S2. Single nuclei suspensions were made directly from frozen tissues. Debris and ruptured nuclei were removed via fluorescence activated cell sorting (FACS), and we tested the transposase reaction for ATAC‐Seq to identify a nucleosome banding pattern (Figure S3). After determining the method preserved chromatin integrity, we proceeded with chromatin accessibility in single nuclei. Combining benign and tumour samples into one UMAP, we showed similarity in the benign pancreatic samples but heterogeneity between PDAC projections (Figure 1A) and characteristic of PDAC tumours.^9^, ^11^, ^15^ While there was some overlap in projections between nuclei from benign specimens and PDAC, the benign and tumour samples generally separated, with 18 694 nuclei identified for the benign specimens and 11 143 nuclei identified for the PDAC specimens (Figure 1B). We annotated eight cell clusters in the benign and tumour combined UMAP through an integrative analysis with the RNA part of snMultiome data for tumour and adjacent control tissues collected from an additional patient (Figures S4–S6), of which the cluster were annotated by expression of literature‐supported markers (Figure S4). Marker genes of each snATAC‐Seq cluster were predicted based on differential promoter openness (Figure S5B; see Table S2 for a full list of the predicted markers). For each snATAC‐Seq cluster, we annotated each cell type by cross‐referring its open chromatin markers to RNA markers for each cell type identified from snMultiome RNA data, calculated overrepresentations, and used that information to transfer the cell annotation at cluster level (Figure S5C). Representative marker genes from each of the snATAC‐Seq clusters were then validated by gene expression in the snMultiome RNA data (Figure S5D). We further validated the annotation through inspecting the positional relationship of the clusters from the snMultiome ATAC when aligned with those from the snATAC‐Seq dataset (Figure S6). Similarly, we validated the annotation with a published scATAC‐Seq dataset derived from freshly acquired nuclei suspensions (Figure S7).^20^

The annotations for the snATAC‐Seq data are summarised in Figure 1C. The majority of nuclei (53.0%) clustered into the acinar cell group (Figure 1D), aligning with literature showing acinar cells constitute approximately 50% of cells in healthy pancreas. Benign samples are predominated by acinar and ADM cells, while myeloid cells are specific to tumour samples (Figure 1E,F). We conducted a Fisher's exact test on the count of each cell type in both benign and tumour samples, yielding *p*‐values consistently less than 5.3e‐25. These findings show the unique cellular composition of the PDAC TME compared to benign pancreas identified by snATAC‐Seq.

### 3.2 Open chromatin provides insight into the regulome and cellular function in benign and PDAC specimens

Chromatin accessibility can be used to infer transcription factor activity, gene promoter activity and therefore predicted molecular functions. Utilising chromVAR,^39^ we identified differential binding of transcription factors across cell clusters (Figure 2A). In this analysis, acinar cells were enriched with T‐cell factor/lymphoid enhancer factor 3 and 12 (TCF3 and TCF12) and Nuclear Receptor Subfamily 5 Group A Member 2 (NR5A2), transcription factors responsible for development and maintenance of the exocrine pancreas.^45^ In the ADM cluster, members of the TEA domain (TEAD) transcription factor family (TEAD1–4) have increased activity compared to the acinar cluster. TEAD transcription factors target SRY‐Box Transcription Factor 9 (SOX9),^46^ which drives ADM.^26^ The ductal cell transcription factor activity was primarily driven by malignant ductal cells characterised by FOS/JUN activity, which comprise the activator protein 1 (AP‐1) transcription factor^47^ and have been implicated in PDAC development.^48^ Endocrine cell transcription factor activity was characterised by the regulatory factor X (RFX) transcription factor, important for development and maintenance of pancreatic beta cell function.^49^ Endothelial cell development is driven through ETS (E26 transformation specific) and ETV (ETS variant) transcription factors^50^ also supported from the ChromVAR analysis. The fibroblast cluster showed activation of SMAD2/3 transcription factor activity, which is important for cancer‐associated fibroblasts development and gemcitabine resistance in PDAC.^51^ The T cell cluster was also characterised by ETS transcription factors 1 and 2. ETS1 and ETS2 have differential functions in human T cells. ETS1 is high in resting T cells, while ETS2 mRNA and protein increase after T‐cell activation.^52^ Transcription factor SPI1 is elevated in the myeloid subset as this is the primary transcription factor driving myeloid lineage cells and promotes monopoiesis over granulopoiesis and lymphoid cell development.^53^ Overall, ChromVAR analysis provides important transcription factor activity data consistent with cell type annotations, again confirming the validity of our cell type annotation for the snATAC‐Seq data sets.

FIGURE 2Regulome and molecular characterisation of cell populations identified in benign and pancreatic adenocarcinoma (PDAC) single nuclear assay for transposase accessible chromatin (ATAC) sequencing (snATAC‐Seq) datasets. (A) Heatmap of top enriched transcription factor motifs activity (row) across cells organised into annotated clusters (column). (B) Heatmap of the ATAC signal at promoters of genes showing cluster‐specific chromatin accessibility. The signal was averaged across all cells within each cluster. Represented genes were shown on the left. (C) Genome browser tracks for ATAC signal across representative genes of each cell type (shown at the top) with differentially accessible regions (including the promoters) highlighted by orange strips. (D) Gene Ontology (GO) enrichment analysis results for genes with promoters differentially accessible in benign samples as compared to tumour samples. (E) GO enrichment analysis results of genes with promoters differentially accessible in PDAC tumour samples as compared to benign. (F) GO enrichment analysis results for genes with promoter specifically accessible in designated cell types (shown at the top) as compared to all others. GO enrichment *p*‐value is indicated by bar color with scale showing the –log *p*‐value to the right. nGenes represents the number of genes under this GO term.

**Figure:**
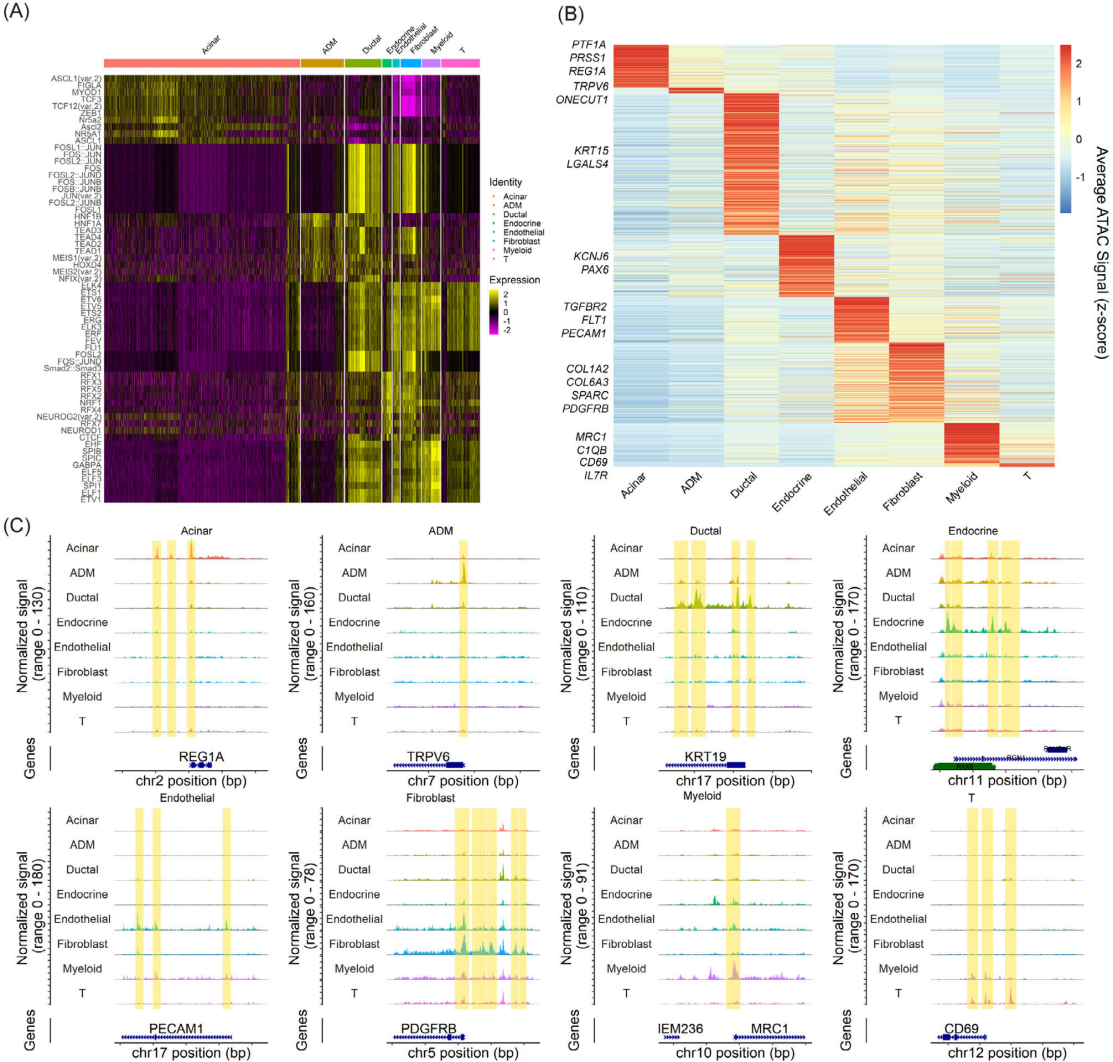

**Figure:**
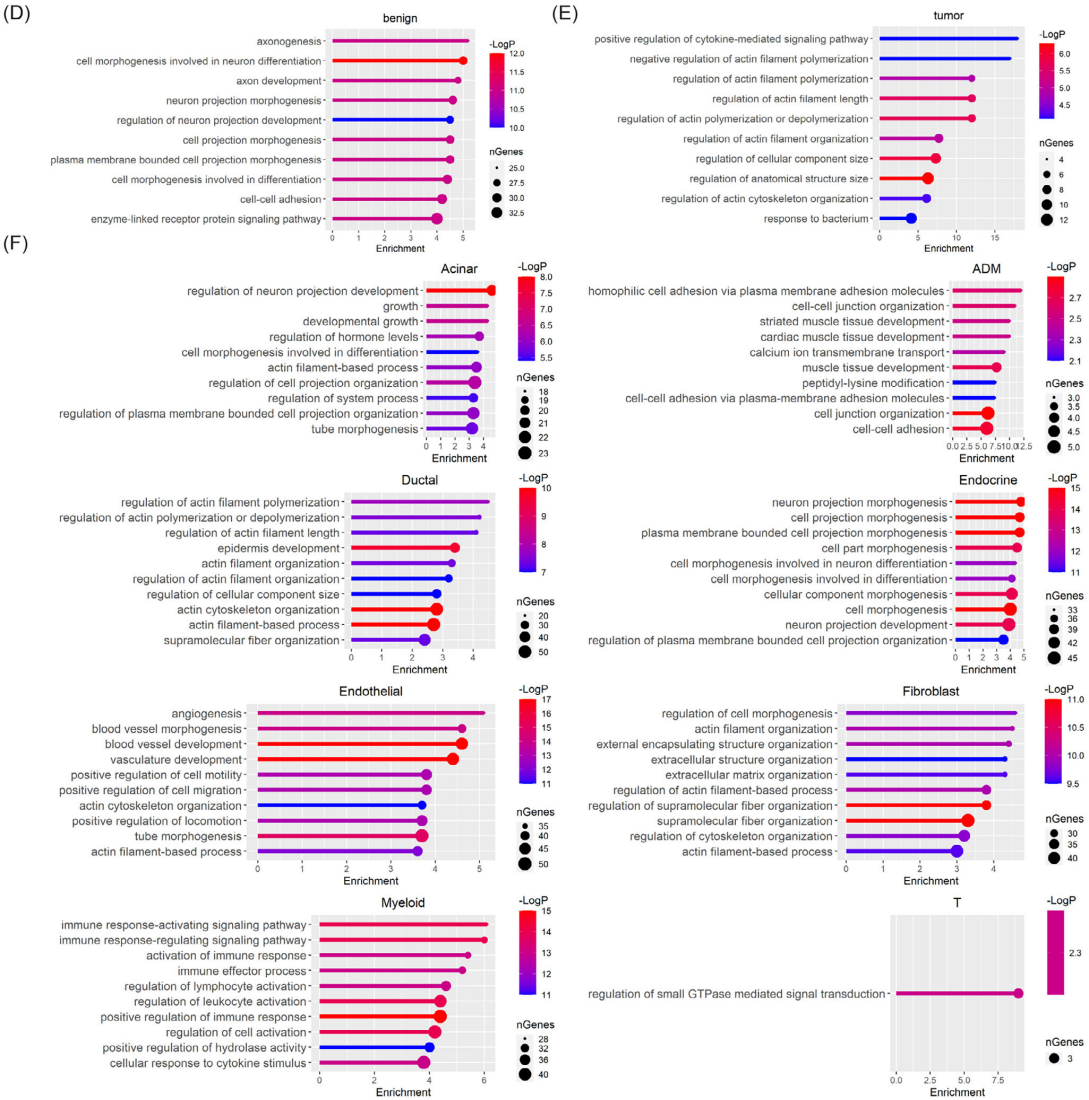

Next, we focused on genes with promoters preferentially opened in one cell type (Table S2). The promoter openings of those genes were first visualised using heatmap (Figure 2B). We next focused on promoter openings at genes known to be markers for each cell type (Figure 2C): regenerating family member 1 alpha (*REG1A*) is expressed by acinar cells.^11^ The ADM group was characterised by transient receptor potential vanilloid member 6 (*TRPV6*) promoter opening, implicated in promoting pancreatic cancer.^54^ Ductal cells in PDAC are characterised as keratin 19 (*KRT19*) positive.^11^, ^15^ Endocrine cells have openings at the promoter for Paired box 6 (*PAX6*), important for development and maintenance of the endocrine pancreas in murine models.^27^ Promoter opening at the platelet and endothelial adhesion molecule 1 (*PECAM1*) promoter is characteristic of endothelial cells.^11^ Platelet‐derived growth factor receptor beta (*PDGFRB*) is characteristic of PDAC fibroblasts.^15^ The myeloid population had opening at the Mannose receptor C‐type 1 (*MRC1*) gene, characteristic of suppressive macrophages prevalent in PDAC.^5^, ^12^ The T‐cell population was characterised by promoter opening at *CD69*, an early activation marker for T cells expressed by T cells in PDAC.^55^

Then, we performed GO enrichment using the genes to identify unique molecular functions between the benign pancreas and PDAC. In the benign specimens, the GO enrichment was predominantly related to the nervous system, including axonogenesis, cell morphogenesis involved in neuron differentiation and axon development (Figure 2D), similar to previous analysis.^7^ These findings demonstrate the strong link between the nervous system and both the endocrine (alpha, beta and delta cells) and exocrine (acinar and ductal cells) pancreas, which are under control of the autonomic nervous system.^56^ In PDAC specimens, the hallmarks of the nervous system–pancreas crosstalk are replaced with functional terms for inflammation (positive regulation of cytokine‐mediated signalling pathway), confirming previous scRNA‐Seq analyses^15^ and regulation of actin filament length, indicative of migratory or dividing cells (Figure 2E).^57^ Previous studies have also shown similar dysregulation of axonal guidance pathways in the progression to PDAC.^7^

We further applied GO enrichment to identify overrepresented molecular pathways in each cluster (Figure 2F; full list is provided in Table S4). Analysis of the acinar cell and endocrine clusters reveals the connection between the autonomic nervous system and pancreas for normal pancreatic development and function.^56^ The acinar cell cluster GO enrichment of regulation of neuron projection development contains the gene *GFI1*, which is known for its function in development of acinar cells.^58^ Furthermore, *FGF9* and *FGF10*, genes recently shown to be involved in pancreatic development^59^ and previously known to be important for neuronal development and maturation^60^ play a role in the GO pathway developmental growth for the acinar cell cluster. In the endocrine cluster, the GO pathway neuron projection morphogenesis is characterised by *Pax6*, a gene responsible for development of the pancreas and other neuroectodermal structures,^61^ *ISL1*, a gene important for beta cell function,^62^ and *ITGB1*, a gene coding for an integrin critical for islet cell development in the pancreas^63^ and for neuronal cell migration.^64^ ADM GO enrichment includes cell–cell junctions and adhesions, representing the transition of acinar cells to a more ductal‐like phenotype.^15^ In the ADM GO enrichment, *PCDH1*, a negative prognostic indicator in PDAC^65^, ^66^ is one of the genes for homophilic cell adhesion via plasma membrane adhesion molecules. Also, interestingly, the gene *HEG1*, which codes for a membrane protein that may be targeted in mesothelioma,^67^ plays a role in the GO enrichment for cell–cell junction organisation. The ductal cells, which were primarily composed of tumour cells, have GO enrichment related to actin filaments.^57^ Specifically, the genes *CAPG* ^68^ and *VASP*,^69^ which are related to metastasis in gastrointestinal cancers are represented in the regulation of actin polymerisation GO biological process identified as a function of the ductal cells. The endothelial cells function in blood vessel and vasculature development. In the endothelial cell cluster, the angiogenesis GO pathway was characterised by genes *NOS3*, important in VEGF‐induced angiogenesis^70^ and implicated as a therapeutic target in PDAC,^71^ and *FGF1*, a known inducer of angiogenesis.^72^ The fibroblast cluster showed expected functions of extracellular matrix and structure organisation,^11^ which were characterised by collagen genes (*COL1A2*, *COL5A1*, *COL8A1*, *COL16A1*). Interestingly, the fibroblast GO enrichment showed functions of regulating cell morphogenesis. Cancer‐associated fibroblasts can activate epithelial to mesenchymal transition (EMT) in pancreatic cancer,^73^ and the fibroblast GO biological pathway was also characterised by *SPARC* expression, which promotes tumour cell invasiveness in triple negative breast cancer when released by fibroblasts.^74^ As previously shown,^75^ T cell activation involves regulation of GTPase mediated signal transduction. The gene *ARHGAP15* was involved in the GO biological process for T cells, and previous studies have shown that different isoforms of ARHGAP15 may play different roles in T cell activation.^76^ For the myeloid cluster GO enrichment, activation of immune response was characterised by the inflammasomes (*NLRP1*, *NRLP3* and *AIM2*), which can activate inflammation in myeloid cells^77^ but may cause immunosuppressive T cells in PDAC.^78^ This function of myeloid activation of the immune system may be contrary to previous reports of myeloid cells in PDAC having immunosuppressive effects.^2^, ^79^ Interestingly, the myeloid GO biological process, cellular response to cytokine stimulus, was characterised by genes *MRC1* and *CSF2RA*, which are commonly associated with more immunosuppressive phenotypes in myeloid cells,^5^, ^11^, ^12^, ^38^ demonstrating that the myeloid cells responding to cytokine stimuli may be reprogrammed to an immunosuppressive phenotype. These findings suggest a spectrum of myeloid effector states in the PDAC TME, which should be further stratified. Taken together, these findings represent snATAC‐Seq as a powerful tool for cellular heterogeneity delineation, regulome characterisation and molecular function inference from frozen pancreatic tissue.

### 3.3 High myeloid cell activity is characteristic of PDAC tumours but does not predict patient survival

**FIGURE 3:**
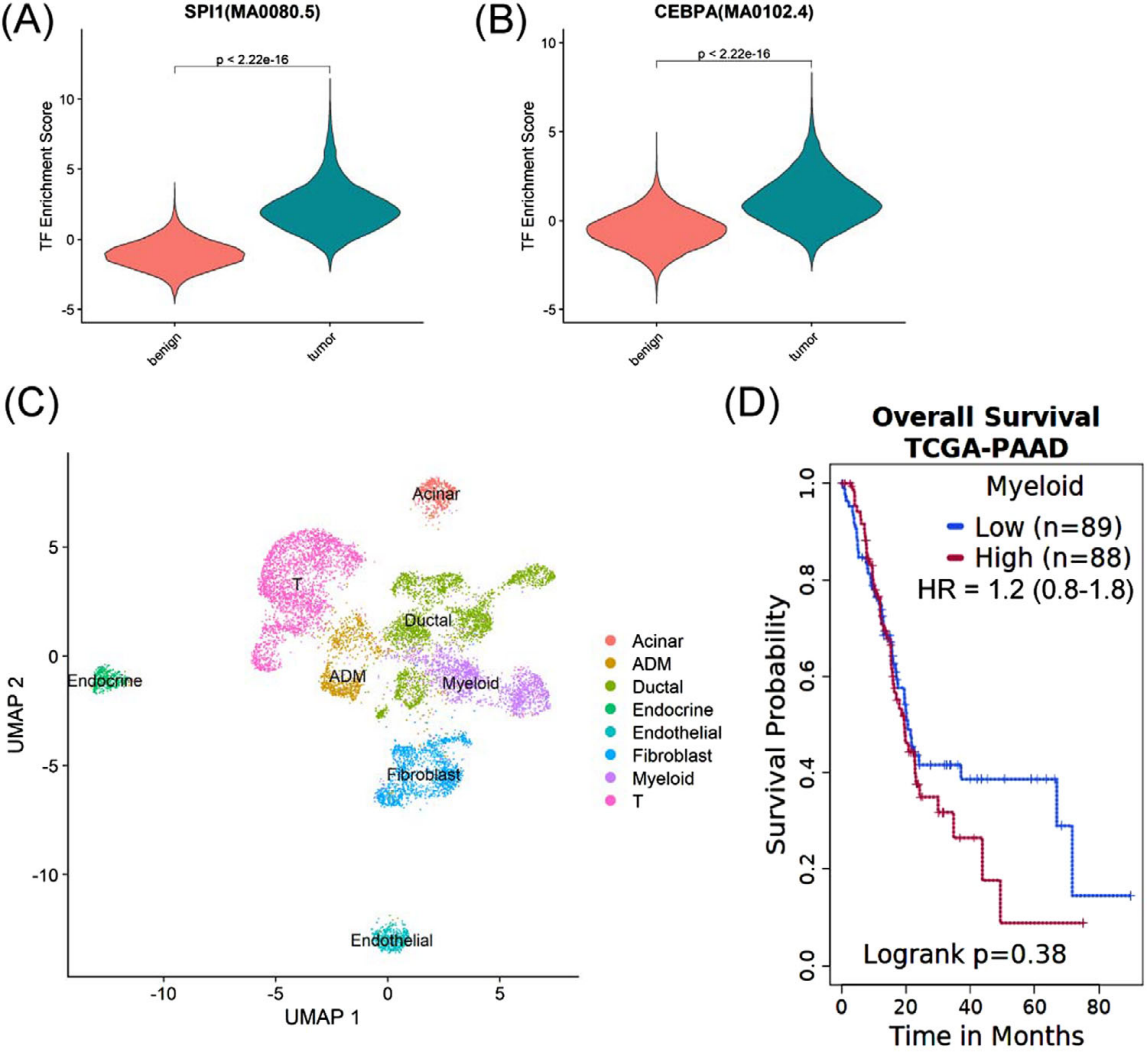
Myeloid populations are higher in pancreatic adenocarcinoma (PDAC) tumour than benign pancreas. (A) Violin plot for transcription factor activity corresponding to SPI1 motif for benign and PDAC tumours from ChromVAR analysis on single nuclear assay for transposase accessible chromatin (ATAC) sequencing (snATAC‐Seq) data. The mean comparison p‐value was calculated with wilcox.test method. (B) Violin plot for CEBPA motif. (C) Re‐clustering and uniform manifold approximation and projection (UMAP) visualisation of cell type among PDAC tumours datasets. (D) Kaplan–Meier survival curve for ‘The Cancer Genome Atlas (TCGA)‐pancreatic adenocarcinoma (PAAD)’ patients sorted by overall expression of myeloid signature genes identified from snATAC‐Seq analysis.

Given the importance of myeloid cells in PDAC and inherent challenges with characterising these highly plastic cells, we next sought to determine if snATAC‐Seq could give insight into myeloid cells. First, we determined that ChromVAR‐inferred binding of transcription factors important for driving myeloid cell development (such as SPI1 and CEBPA) were more significantly more prevalent in tumour than benign pancreata (Figures 3A,B). These data further demonstrate the findings in Figure 1, in which only 24 nuclei representing myeloid cells (1.6% of the total myeloid population) were identified in the benign pancreata, consistent with previous analyses showing limited myeloid cells in normal pancreas.^12^, ^20^ Given, the low number of myeloid nuclei identified in the benign pancreata, we further characterised the myeloid subpopulations in the PDAC TME alone. To do this, we re‐clustered only the PDAC samples and annotated all the cell populations. We then focused on myeloid cells in the tumour samples (Figure 3C) and identified the top 10 genes with differential promoter openings in this cluster relative to other clusters (Figure 3D). Stratifying patients by high or low myeloid gene signatures using the Survival Genie application with the pancreatic adenocarcinoma (PAAD) dataset from The Cancer Genome Atlas (TCGA), we found no difference in overall survival between patient populations based on high or low expression of the tumour myeloid cell gene signature (Figure 3D). Since previous studies showed differences in disease progression or patient prognosis based on myeloid subsets in the tumour,^2^, ^5^, ^9^, ^79^ we next sought to stratify the myeloid cells.

### 3.4 Unique myeloid cell populations exist in PDAC tumour and have distinct functions

**FIGURE 4:**
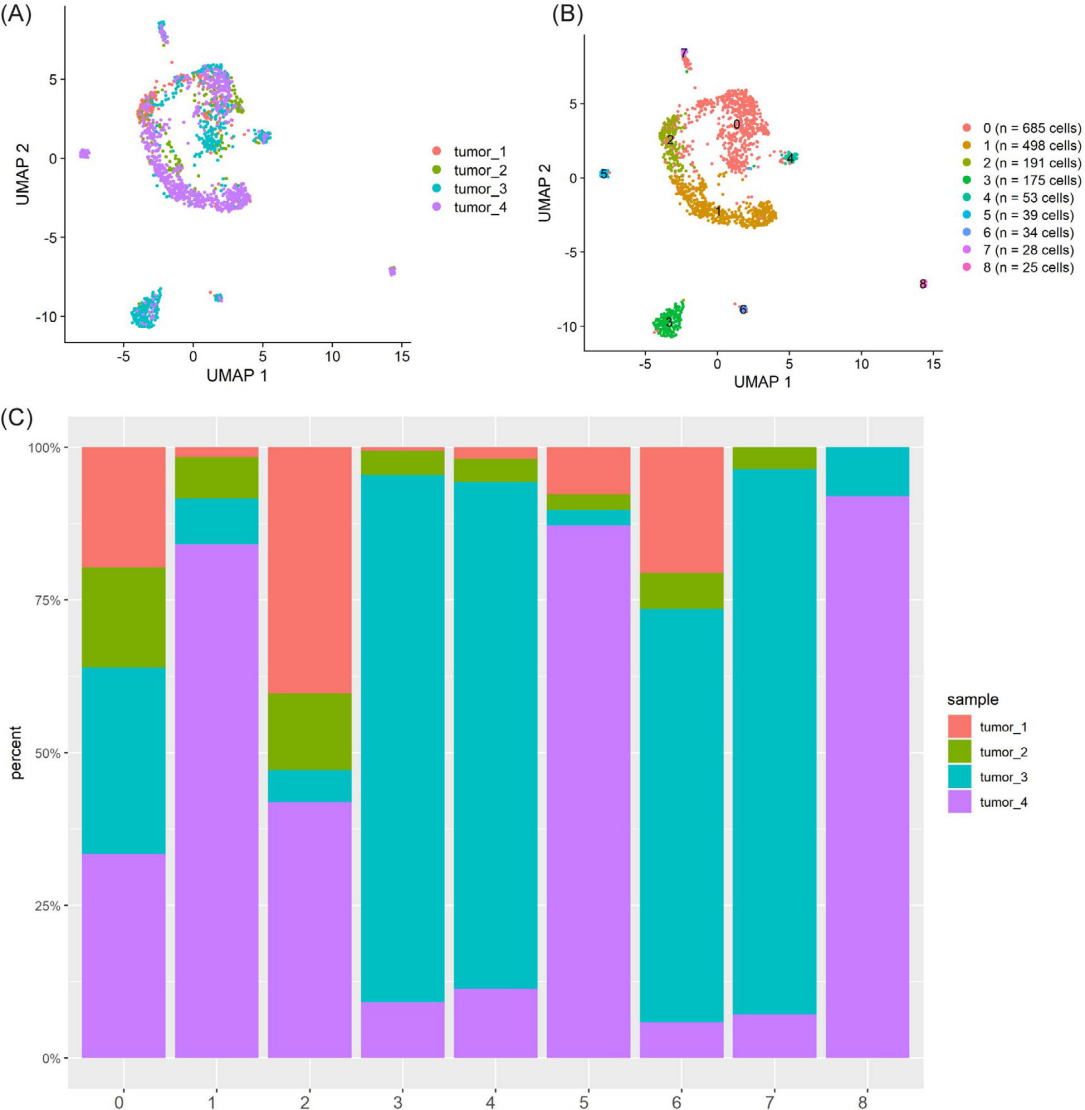
Myeloid subclusters in pancreatic adenocarcinoma (PDAC) tumours. (A) Uniform manifold approximation and projection (UMAP) visualisation of the PDAC tumour dataset only for the myeloid populations. (B) UMAP visualisation of the identified subclusters. (C) Distribution of cells from each PDAC tumour to the subclusters identified in (B).

Observing no survival difference between groups with high and low myeloid signature gene expression, we further delineated the myeloid cluster into subclusters corresponding to several types of myeloid cells.^11^ We extracted the myeloid cell populations from the PDAC tumour samples (Figure 4A), performed re‐clustering analysis and identified nine subclusters (Figure 4B). Most cells grouped into subclusters 0–4, which had contributions from all the assayed tumours (Figure 4B). The four smaller clusters of nuclei (subclusters 5–8) comprising less than 50 nuclei each and not represented in all tumours were excluded from further analysis (Figure 4B). Our snATAC‐Seq analysis revealed each patient contributed differently to each myeloid subcluster (Figure 4C) consistent with the concept of tumour immune cell heterogeneity.^9^, ^11^ Overall, analysis of myeloid cell populations from frozen PDAC tumours identified five major myeloid cell populations for further analysis type and function.

**FIGURE 5:**
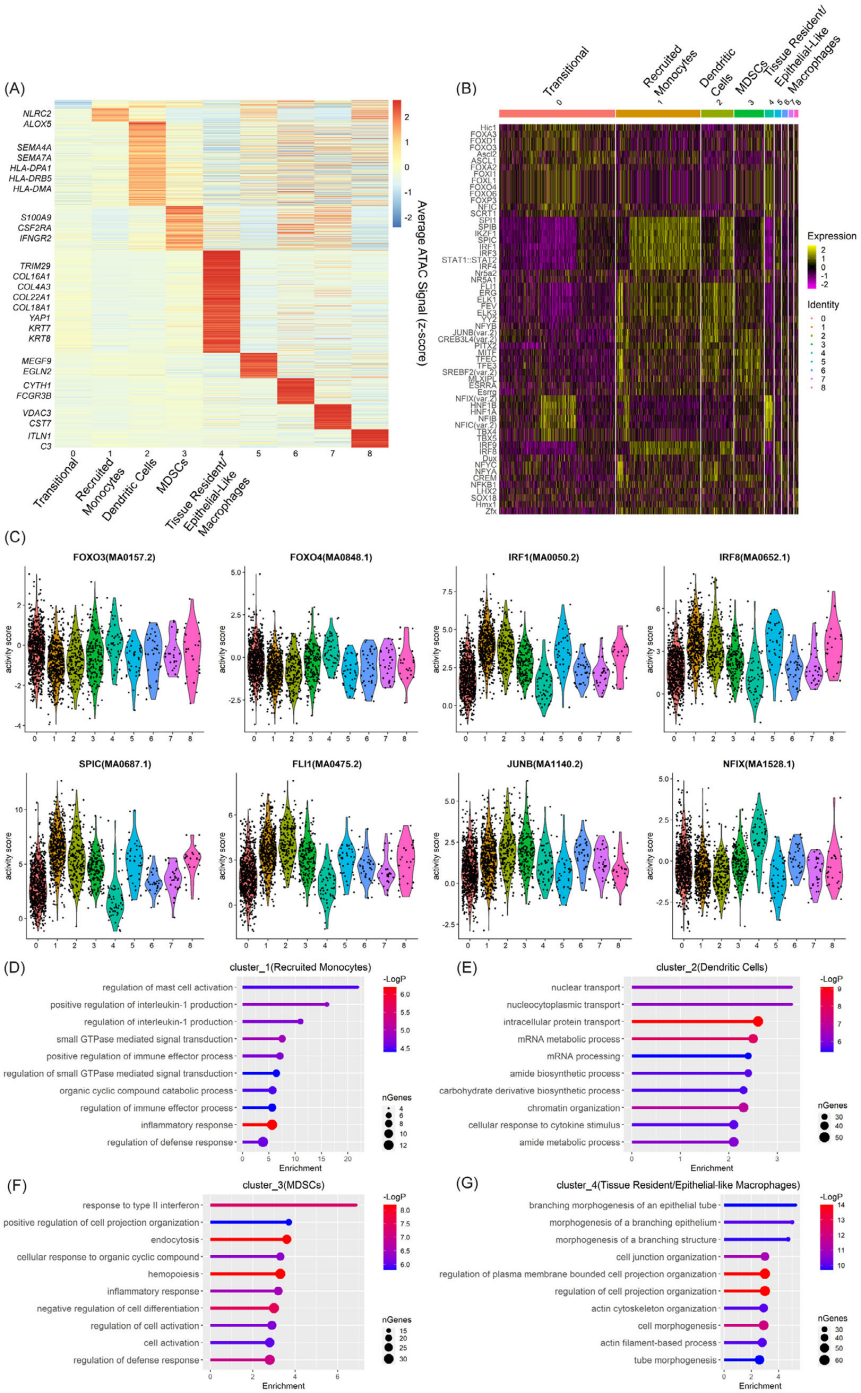
Characterisation of myeloid subclusters in pancreatic adenocarcinoma (PDAC) tumours. (A) Heatmap of the assay for transposase accessible chromatin (ATAC) signal at promoters showing cluster‐specific chromatin accessibility within the myeloid subclusters. The signal was averaged across all cells within each subcluster. Represented genes were shown on the left (*p* < .01). (B) Heatmap of top enriched transcription factor motifs activity (row) across cells organised into subclusters from myeloid cells (column). (C) The violin plot showing ChromVAR‐inferred transcription factor activity score for selected motifs across subclusters. (D–G) Gene Ontology (GO) enrichment analysis results for genes with promoters preferentially open in myeloid subcluster of 1 (D), 2 (E), 3 (F) and 4 (G). GO enrichment *p*‐value is indicated by bar color with scale showing the –log *p*‐value to the right. nGenes represents the number of genes under this GO term.

To further characterise the myeloid subpopulations, we determined their unique promoter openings, transcription factor activity and functional profiles. We first identified promoter regions preferentially open in each subcluster (Figure 5A). We noted no promoter upregulation in subcluster 0, suggesting the cells might be in a transitional state. In myeloid subcluster 1, we identified promoters for monocyte‐related genes (*ALOX5* and *NLRC4*). Arachidonate 5‐lipoxygenase (*ALOX5*) is important for generating leukotrienes from arachidonic acid,^31^ while NLR family CARD domain containing 4 (*NLRC4*) is a monocyte marker^32^ important for activation of the innate immune response.^80^ Myeloid subcluster 2 is characterised by semaphorin 4A (*SEMA4A*), a marker for dendritic cell activation of T cells,^33^, ^34^ and semaphorin 7A (*SEMA7A*), which promotes dendritic cell migration.^35^ Myeloid subcluster 3 was identified as MDSCs based on S100 calcium binding protein A9 (*S100A9*), colony stimulating factor 2 receptor subunit alpha (C*SF2RA*) and interferon gamma receptor 2 (*IFNGR2*) expression.^11^, ^38^ Myeloid subcluster 4 was characterised by collagen expression (*COL16A1*, *COL4A3*, *COL22A1*, *COL18A1*) and Yes1‐associated transcription regulator (*YAP1*), all TRM markers in murine PDAC.^5^ Myeloid subcluster 4 also expresses keratin 7 and 8 (*KRT7* and *KRT8*) aligning with epithelial‐like myeloid cells identified by scRNA‐Seq in PDAC.^11^

ChromVAR analysis of the transcription factor activity between the myeloid clusters was mostly distinct with some overlap as expected from highly plastic myeloid populations (Figures 5B,C). Myeloid subcluster 0 is characterised by FOXO transcription factor activity, which is important in maintenance of haematopoietic stem cells^81^ and may be important in peritoneal TRMs.^82^ These cells may be a transitional state of myeloid cell as pancreatic TRMs can be replaced by stem cells upon damage to the pancreas.^83^ Myeloid subclusters 1 (recruited monocytes) and 2 (dendritic cells) were characterised by IRF1 transcription factor activity, a promoter of anti‐tumour immunity in conventional dendritic cells.^84^ IRF8 binding is also higher in myeloid subclusters 1 (recruited monocytes) and 2 (dendritic cells) and promotes differentiation of monocytes and dendritic cells.^85^, ^86^ In myeloid subcluster 1 (recruited monocytes), the SPIC transcription factor, promoting anti‐inflammatory function in monocytes^87^ and angiogenic tumour‐associated macrophages,^88^ is characteristic. In contrast, FLI1 transcription factor in myeloid subcluster 2 (dendritic cells) is elevated, which drives development of phagocytic monocytes in mice.^89^ Myeloid subcluster 3 (MDSCs) has higher JunB and lower IRF8 activity. While JunB can drive macrophage activation,^90^ it also drives MDSCs.^91^ IRF8 activity, which suppresses MDSC development, is lower in myeloid subcluster 3 (MDSCs).^92^ The transcription factor activity for myeloid subcluster 4 (tissue resident/epithelial‐like macrophages) is similar to the activity for ADM, resembling the epithelial‐like myeloid cells shown by scRNA‐Seq in PDAC.^11^ Myeloid subcluster 4 also has elevated FOXO4 activity, whose activity has been shown to downregulate genes in peritoneal TRMs after feeding.^82^ Furthermore, NFIX, elevated in myeloid subcluster 4 (tissue resident/epithelial‐like macrophages), can drive fibrosis in macrophages in murine models of muscular dystrophy.^93^ Taken together, these findings give insight into specific regulome of each myeloid subset.

We next performed GO enrichment analysis to understand the molecular function of the myeloid cells (Table S5). We show that recruited monocyte (myeloid subcluster 1) function is related to mast cell activation (*IL4R*, *NECTIN2*), IL‐1b production (*TYROBP*, *NLRP3*) and positive regulation of immune effector processes (*IL1B*, *IL4R*, *NLRP3*) (Figure 5D), suggesting monocytes are recruited and activated. Dendritic cell (myeloid subcluster 2) activity was related to nuclear, nucleocytoplasmic and intracellular transport (*BCL3*, *STAT3*, *CBLB*) (Figure 5E), related to the function of dendritic cell antigen processing and presentation.^94^ MDSC (myeloid subcluster 3) GO enrichment showed response to type II interferon (*IFNGR*, *CXCL16*, *IRF1*, *TLR2*) (Figure 5F). Type II interferon response is linked to recruited monocytes^95^ and MDSCs.^38^ Tissue resident/epithelial‐like macrophage (myeloid subcluster 4) activity demonstrated actin cytoskeletal organisation (*ACTG1*, *ACTN1*, *MYO1F*, *MYO1A*, *MYO1C*) (Figure 5G), which may indicate cell division characteristic of TRMs in PDAC murine models^5^ and exacerbated with chemotherapy.^43^ Overall, our refined snATAC‐Seq analysis delineated cellular heterogeneity among myeloid cells in PDAC tumours and identified molecular pathways overrepresented in each subpopulation.

### 3.5 Genotypic signatures of dendritic cells and tissue‐resident macrophages may predict survival in PDAC patients

**FIGURE 6:**
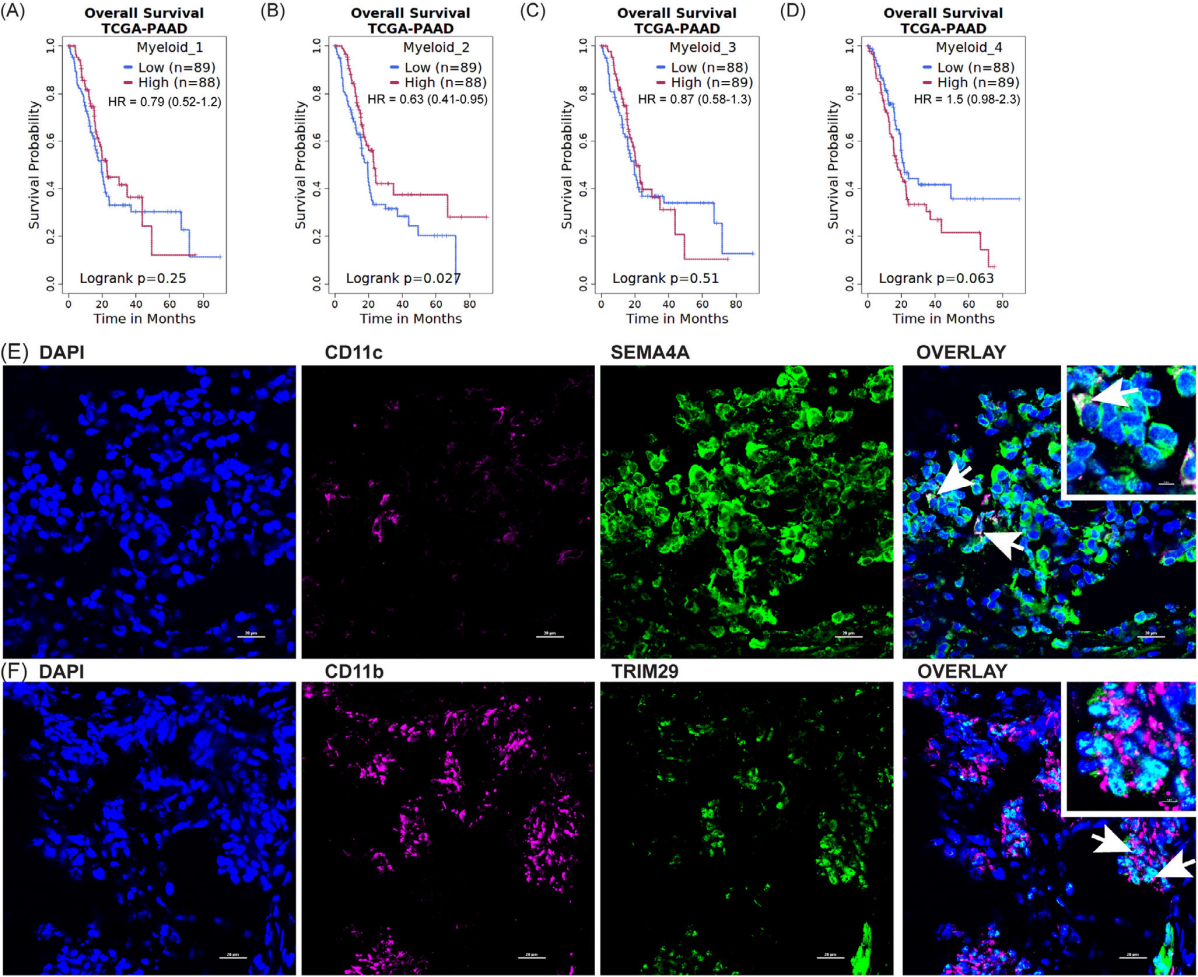
Signature genes from myeloid subset predict overall survival in The Cancer Genome Atlas (TCGA)‐pancreatic adenocarcinoma (PAAD) dataset. (A–D) Kaplan–Meier survival curve for ‘TCGA‐PAAD’ patients sorted by overall expression of signature genes for each myeloid subcluster: recruited monocyte (A), dendritic cells (B), myeloid‐derived suppressor cell (MDSC) (C) and tissue resident/epithelial‐like macrophage (D). (E) Immunofluorescent (IF) staining for dendritic cell markers CD11c and SEMA4A in tumour 4. (F) IF staining for tissue resident/epithelial‐like macrophage markers CD11b and TRIM29 in tumour 3. Images taken at 60× (20 μm scale). White arrows indicate co‐stained cells. Inset indicates co‐stained cells (5 μm scale).

Having annotated the identity and functions of the myeloid subclusters, we next endeavored to determine if these myeloid subpopulations impacted survival. To assess a large patient cohort, we explored gene signatures for myeloid subpopulations in the TCGA‐PAAD dataset. Using the Survival Genie^41^ application, the signature genes for myeloid subcluster 1 (recruited monocytes) showed no survival difference, likely due to the mixed nature of this subset, where classical (pro‐inflammatory) and nonclassical (anti‐inflammatory) monocytes may have disparate effects on survival (Figure 6A). In contrast, high expression of the myeloid subcluster 2 (dendritic cell) signature in the tumour improved patient survival in the TCGA‐PAAD cohort (Figure 6B), which has been similarly reported for circulating dendritic cells.^36^ We demonstrate no difference in survival based on the myeloid subcluster 3 (MDSC) gene signature (Figure 6C). While not statistically significant in the TCGA‐PAAD cohort, there is a trend (*p* = .063) towards improved survival in patients with lower gene signature for myeloid subcluster 4 (tissue resident/epithelial‐like macrophages) (Figure 6D). We then compared the significant gene signatures for myeloid subclusters 2 (dendritic cells) and 4 (tissue resident/epithelial‐like macrophages) in other gastrointestinal malignancies, including colon adenocarcinoma (TCGA‐COAD), cholangiocarcinoma (TCGA‐CHOL) and stomach adenocarcinoma (TCGA‐STAD) and saw no significant differences in survival (Figure S8). These findings support previous findings demonstrating that the prognostic significance of the myeloid cell infiltrate is unique to PDAC.^11^

Since we identified significant improvement in survival for the cluster 2 signature (dendritic cells) and a trend towards significant detriment to survival for the cluster 4 signature (tissue resident/epithelial‐like macrophages) in the TCGA‐PAAD dataset, we performed fluorescent immunohistochemistry to qualitatively identify co‐staining of signature genes that may be important for the function of these cells, *SEMA4A* and *TRIM29*, respectively, with known myeloid markers. SEMA4A, which improves the ability of dendritic cells to activate T‐cell responses^33^, ^34^ and was elevated in our gene signatures for subcluster 2, was co‐stained with CD11c, a dendritic cell marker (Figure 6E). TRIM29 is implicated in the immunosuppressive functions of macrophages,^96^ and we also identified TRIM29, which can function in the nucleoplasm,^97^ expression in the nuclei of CD11b‐expressing myeloid cells (Figure 6F). These data qualitatively validate the expression of SEMA4A and TRIM29 with known myeloid cell markers, CD11c and CD11b, respectively. Taken together, these data indicate that snATAC‐Seq analysis can provide signature genes of interest for myeloid cells, such as infiltrating dendritic cells (myeloid subcluster 2) and tissue resident/epithelial‐like macrophages (myeloid subcluster 4), which can be further explored in the preclinical and clinical settings.

## 4 DISCUSSION

In this study, we demonstrate snATAC‐Seq can determine the cell compositions of frozen benign pancreas and PDAC samples. Assaying the accessible chromatin regions provides rich information on regulome landscape including transcription factor activities and gene promoter openness. These data can be utilised to determine cell type and infer molecular functionality. In PDAC specifically, previous studies have demonstrated that ATAC‐Seq can be used to determine differentially accessible chromatin peaks in Epcam^+^ epithelial cells sorted from surgically resected PDAC specimens. These peaks can then be used to predict patient disease‐free survival.^98^ Another study utilising scATAC‐Seq from freshly acquired benign pancreatic, normal adjacent tissue and PDAC specimens demonstrated that the KRAS mutation locks open an enhancer network in human PDAC that is similar to that found in murine PDAC models.^20^ Furthermore, another technique to look at open chromatin regions (single cell COOL‐seq) has been used in PDAC specimens to demonstrate specific transcription factor binding motifs that may be important in pancreatic cancer progression.^7^ To add to this body of literature, we employed snATAC‐Seq to explore the intricate regulome landscape of the diverse myeloid populations present in PDAC tumours.

We demonstrated the composition differences among cell populations presented in benign pancreata and PDAC and validated our cell type annotations with snMultiomics and previously published scATAC‐Seq,^20^ adding rigour to our approach. snATAC‐Seq was also used to compare the function of the benign pancreas, related to the pancreatic connection to the autonomic nervous system,^56^ to the function of the cells in the PDAC tumour, which is related to infiltration of inflammatory cells similar to previous reports.^15^ Our GO enrichment determined the function of individual cell populations with support from the literature: the acinar and endocrine cells had links to the nervous system,^1^, ^56^ due to their functions related to neuron projection. The fibroblasts were shown to regulate cell morphogenesis such as previous studies showing fibroblasts drive EMT in PDAC tumour cells.^73^ We similarly identified functions of myeloid subsets, including immune response activation and regulation of lymphocyte and leukocyte activation. These findings were counter to previous studies showing myeloid subsets are primarily immunosuppressive in PDAC,^2^, ^11^, ^79^ prompting further inquiry.

While the current study used four patient tumour samples to characterise myeloid subpopulations, the limited number of patients precluded associations with prognosis. Therefore, we used the determined gene signatures within a larger dataset to determine the influence of the ATAC defined unique populations in the myeloid compartment on survival. Unlike previous studies of infiltrating monocytes, we saw no correlation to overall survival based on expression of signature genes from monocyte^2^ or MDSC^79^ subclusters. Importantly, the previous studies measured monocytes^2^ and MDSCs^79^ in circulation, which may explain the differences in findings. The gene signature for dendritic cells predicted better overall survival, similar to previous reports for circulating dendritic cells.^36^ Previous studies also show infiltrating dendritic cells promote a T cell response in PDAC,^99^ and our gene signature showed expression of *SEMA4A*, which promotes T‐cell activation.^33^, ^34^ Furthermore, there was a trend towards worse overall survival in patients with a tissue resident/epithelial‐like macrophages gene signature, supporting studies showing TRMs responsible for PDAC progression in murine models^5^ and chemotherapy resistance.^43^ Similar to previous studies,^5^, ^11^ the tissue resident/epithelial‐like signature had higher collagen expression. We also uniquely demonstrated *TRIM29* expression in the tissue resident/epithelial‐like signature, which can drive macrophage immunosuppressive features.^96^ The TRM/epithelial‐like population was also like the epithelial‐like macrophage population shown by scRNA‐Seq of PDAC,^11^ suggesting that TRMs and epithelial‐like macrophages may arise from a similar cell population in PDAC tumours. The diverse populations of myeloid cells shown in this study are similar to scRNA‐Seq studies showing unique populations within the myeloid cluster.^11^

Clinical trials focused on taming myeloid cells in PDAC have only been modestly successful. CCR2 antagonism therapy, which prohibits recruitment of circulating monocytes into the tumour, improved tumour control based on RECIST criteria when combined with standard of care FOLFIRINOX.^3^ However, combining CCR2 antagonism with gemcitabine/nab‐paclitaxel did not improve outcomes and was countered by risk of lung toxicity.^4^ While the CCR2/CCL2 axis may recruit myeloid cells that are hijacked by the tumour to become immunosuppressive,^2^, ^3^ these cells can promote an anti‐tumour response when activated to a pro‐inflammatory state.^95^ Furthermore, studies in murine models have shown TRMs, not monocytes, are the problematic population in PDAC,^5^ but these cells have not been targeted clinically. Additional clinical trials have utilised CD40 agonism to promote antigen presenting cells to stimulate a T cell response,^100^ again with limited efficacy. This may be in part due to dendritic cells also having an immunosuppressive function in tumours by expressing cytotoxic T lymphocyte‐associated protein 4 (CTLA‐4)^101^ or programmed cell death protein 1 (PD‐1),^102^ preventing antigen presentation and T cell activation. Thus, while dendritic cells are critical for T cell activation,^37^, ^99^ overactivation can be detrimental and cause immunosuppression.^37^, ^101^, ^102^ Such observations point to the heterogenous nature of the myeloid cell infiltrate in PDAC, which we demonstrate here. This heterogeneity is driven by the interplay between the many factors comprising the PDAC TME, such as tumour cells, stromal cells, immune cells and soluble factors, which can differently impact myeloid populations. For example, *CSF1–CSF1R* interactions between tumour cells and macrophages,^12^ respectively, are important for activation of macrophages. Importantly, colony stimulating factor 1 (CSF1) has been shown to be important for maintaining TRM populations in PDAC, which are pro‐fibrotic and pro‐tumour,^5^ and may be responsible for therapeutic resistance to standard of care chemotherapy gemcitabine.^43^ PDAC tumour cells can also inhibit development of dendritic cells by release of granulocyte colony stimulating factor (G‐CSF), which inhibits IRF8‐mediated development of dendritic cells in the bone marrow.^36^ Furthermore, pancreatic stellate cells from the PDAC TME drive infiltrating myeloid cells towards a MDSC state via STAT3 signalling, which can in turn inhibit T‐cell function.^103^ In contrast, the combination of C‐C motif chemokine ligand 2 (CCL2) and interferon gamma (IFN‐g) can drive an anti‐tumour phenotype in PDAC myeloid cells, and this is particularly important in the presence of CD40 agonist treatment.^95^ Thus, technologies such as snATAC‐Seq can be useful for understanding the presence and function of myeloid cell subpopulations, which we have shown in the present manuscript. Such an understanding could lead to a more targeted approach towards implementation of myeloid therapies rather than a ‘copy/paste’ or ‘one size fits all’ approach, which has failed to translate immunotherapies that have been successful in other cancers to PDAC.^104^ Further studies utilising snATAC‐Seq may uncover additional myeloid targets for future preclinical and clinical studies, emphasising a bedside to bench to bedside approach.

The current study has several potential limitations. While the current manuscript offers methodology for analysing myeloid populations in PDAC tumour specimens across multiple institutions with diverse patient demographics and treatment regimens, the current study only utilises four patients with benign pancreatic pathologies and four patients with PDAC. Given the limited number of PDAC patients, additional studies are warranted to better understand the myeloid populations, as this limited sample size may not be sufficient to conclusively characterise all potential myeloid subpopulations and their functions given tumour and patient heterogeneity. However, the present method provides a means for assaying frozen samples, which may broaden the available patient cohort by allowing for assessment of banked samples.

## 5 CONCLUSION

Despite a small sample size used in our analysis, this manuscript demonstrates the strength of utilising frozen tumour samples with snATAC‐Seq. Frozen samples can be used to study patients with known outcomes and treatment conditions to tease apart the intricacies of the PDAC TME, particularly the myeloid subset, which can be challenging to study given the plasticity of the cells. Additional studies are warranted to understand how neoadjuvant therapies can affect myeloid subpopulations and how those myeloid subpopulations may in turn affect therapy response. With the implementation of such studies, patient‐specific myeloid targets could be identified to better utilise myeloid therapies in particular patient populations or to implement novel myeloid targets.

## AUTHOR CONTRIBUTIONS

*Conceptualisation*: Hillary G. Pratt, Barbara Szomolay, Thomas Whalley, Sascha Ott, Brian A. Boone and Timothy D. Eubank. *Methodology*: Hillary G. Pratt, Sebastian A. Dziadowicz and Li Ma. *Assays*: Hillary G. Pratt and Sebastian A. Dziadowicz. *Data analysis*: Li Ma, Gangqing Hu, Barbara Szomolay and Thomas Whalley. *Writing*: Hillary G. Pratt, Li Ma and Gangqing Hu. *Supervision*: Brian A. Boone, Timothy D. Eubank and Gangqing Hu.

## CONFLICT OF INTEREST STATEMENT

The authors declare they have no conflicts of interest.

## 6 ETHICS STATEMENT

Ethics was reviewed by Institutional Review Board and approval was obtained from West Virginia University (#1903496995), and all patients signed informed consent.

## Supporting information

Table S1

Table S2

Table S3

Table S4

Table S5

Supplementary Figures

Supporting Information

## Data Availability

All raw sequenced data are publicly available at Gene Expression Omnibus under accession number GSE241896.
