# Supplementary material for: Analysis of single nuclear chromatin accessibility reveals unique myeloid populations in human pancreatic ductal adenocarcinoma
Source: Clin Transl Med. 2024 Mar 1;14(3):e1595. doi: 10.1002/ctm2.1595 (PMC10905544; doi:10.1002/ctm2.1595)
Supplement: Supplementary file 6 — Supplementary Figures [file CTM2-14-e1595-s002.docx]

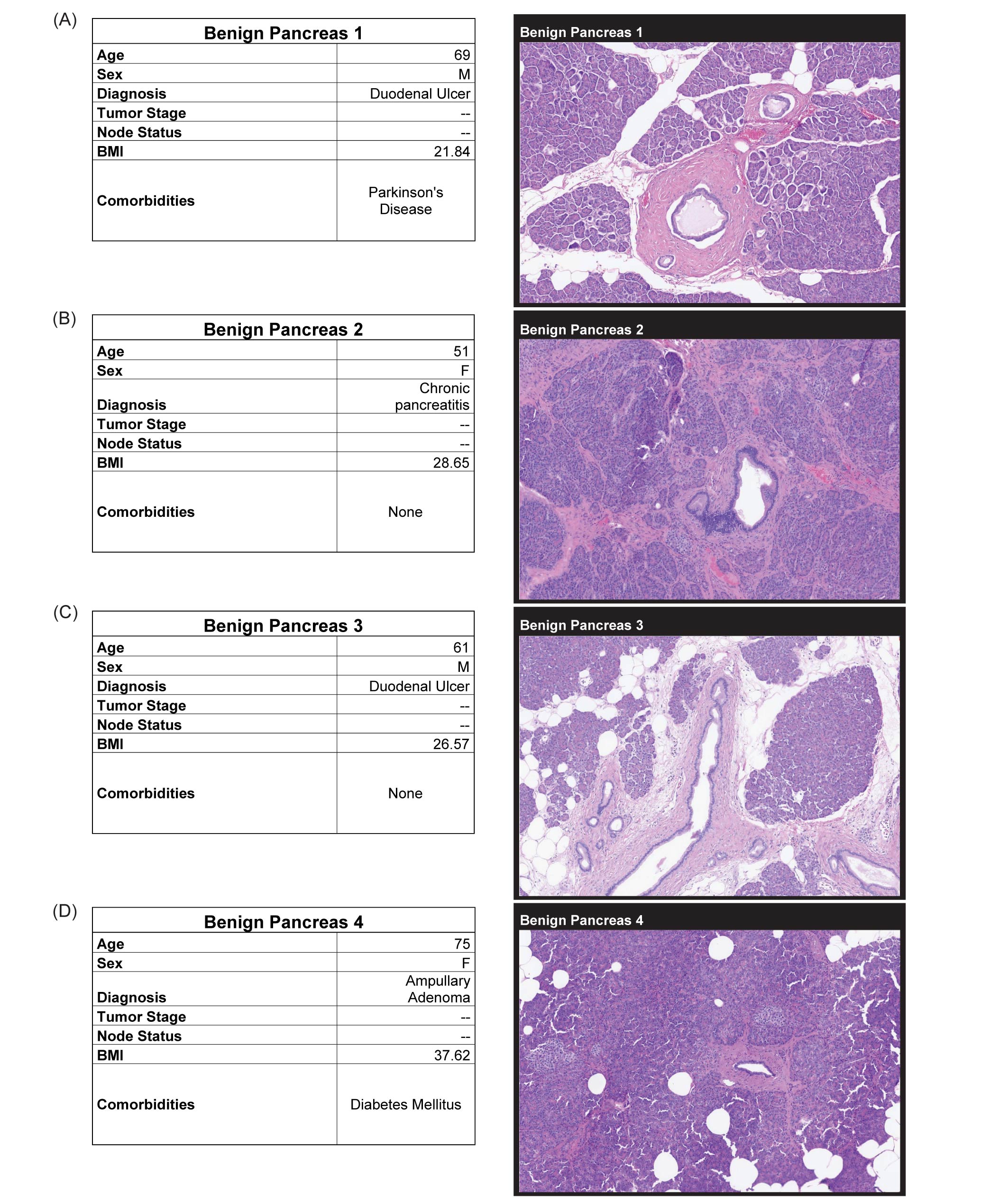


**Supplemental Fig. 1 | Benign pancreatic tissue samples and patient characteristics.** (A) Benign pancreas 1 pathologic diagnosis was duodenal ulcer. Histology shown on the right. (B) Benign pancreas 2 pathologic diagnosis was chronic pancreatitis. Histology shown on the right. (C) Benign pancreas 3 pathologic diagnosis was duodenal ulcer. Histology shown on the right. (D) Benign pancreas 4 pathologic diagnosis was ampullary adenoma. Histology shown on the right.


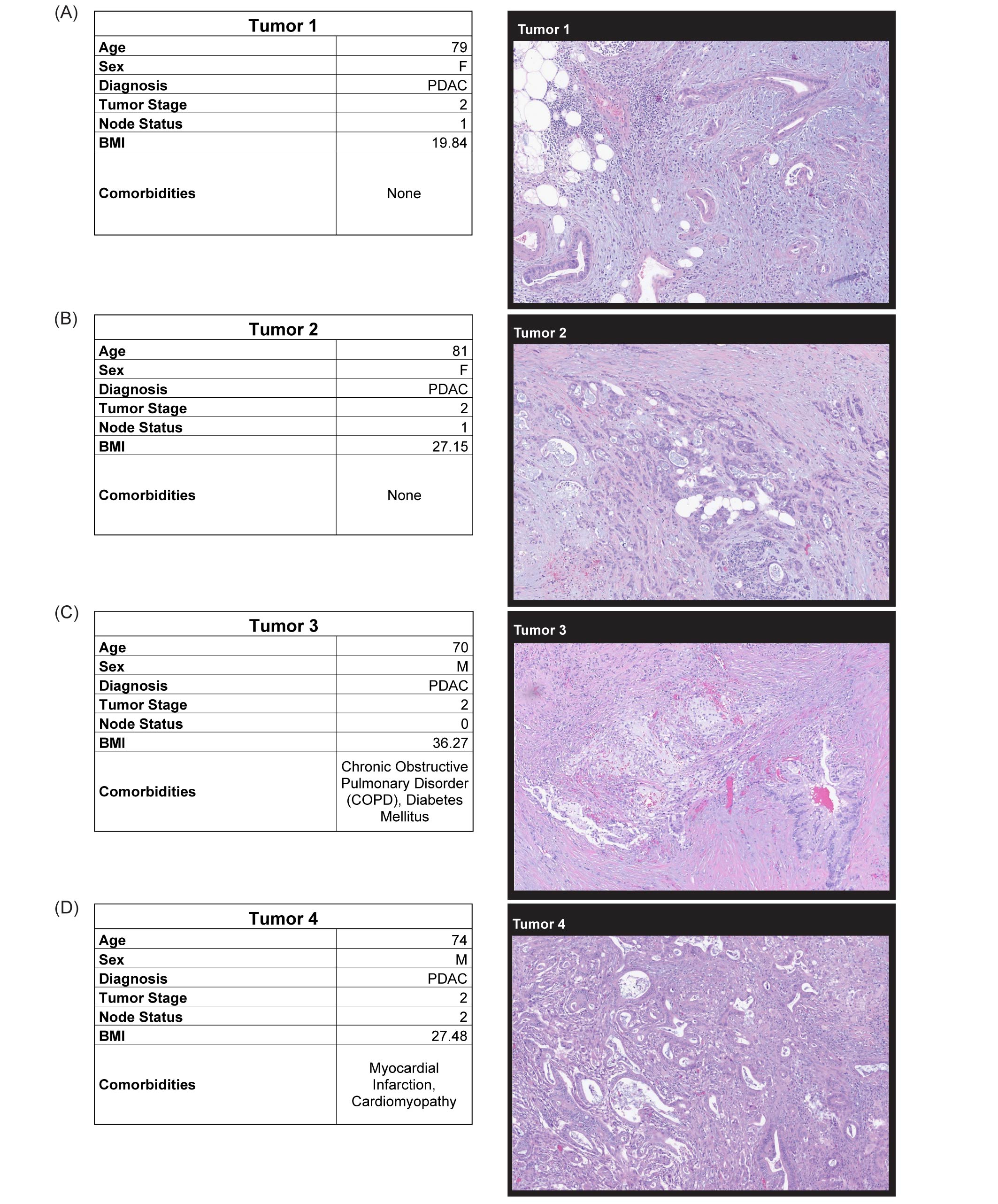


**Supplemental Fig. 2 | PDAC tumor tissue samples and patient characteristics.** (A) Tumor 1 patient characteristics (PDAC) and representative histology. (B) Tumor 2 patient characteristics (PDAC) and representative histology. (C) Tumor 3 patient characteristics (PDAC) and representative histology. (D) Tumor 4 patient characteristics (PDAC) and representative histology.

**
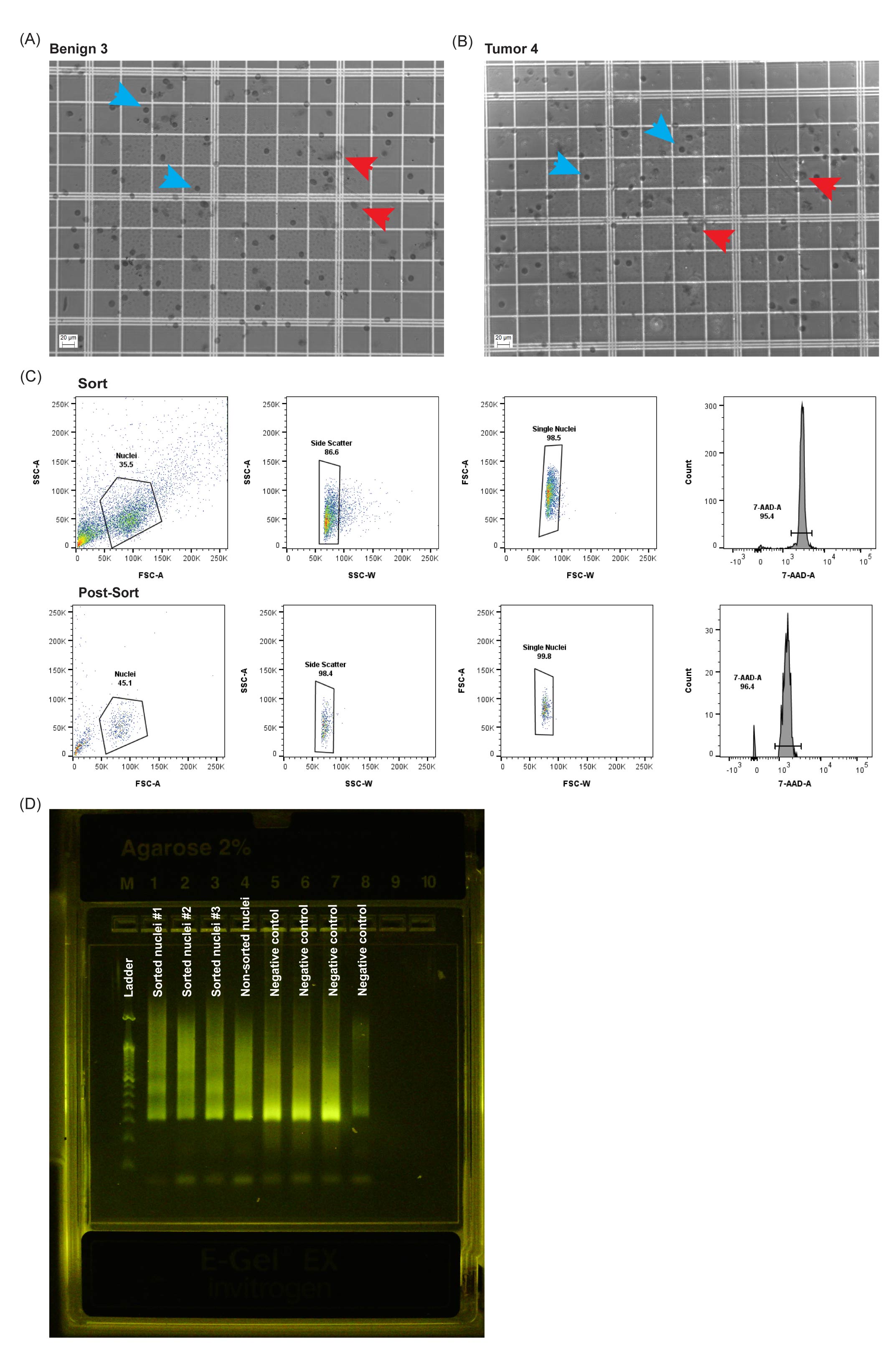
Supplemental Fig. 3 | Nuclei isolation optimization.** (A) Nuclei isolated from benign pancreatic samples stained with Trypan blue. Blue arrow indicates intact nuclei. Red arrow indicates debris to be removed with FACS sorting. (B) Nuclei isolated from benign pancreatic samples stained with Trypan blue. Blue arrow indicates intact nuclei. Red arrow indicates debris to be removed with FACS sorting. (C) Example of sorting and isolation of intact nuclei for transposase reaction. (D) Nucleosome banding for transposase reaction carried out on sorted and unsorted nuclei isolated from PDAC tumor tissue.


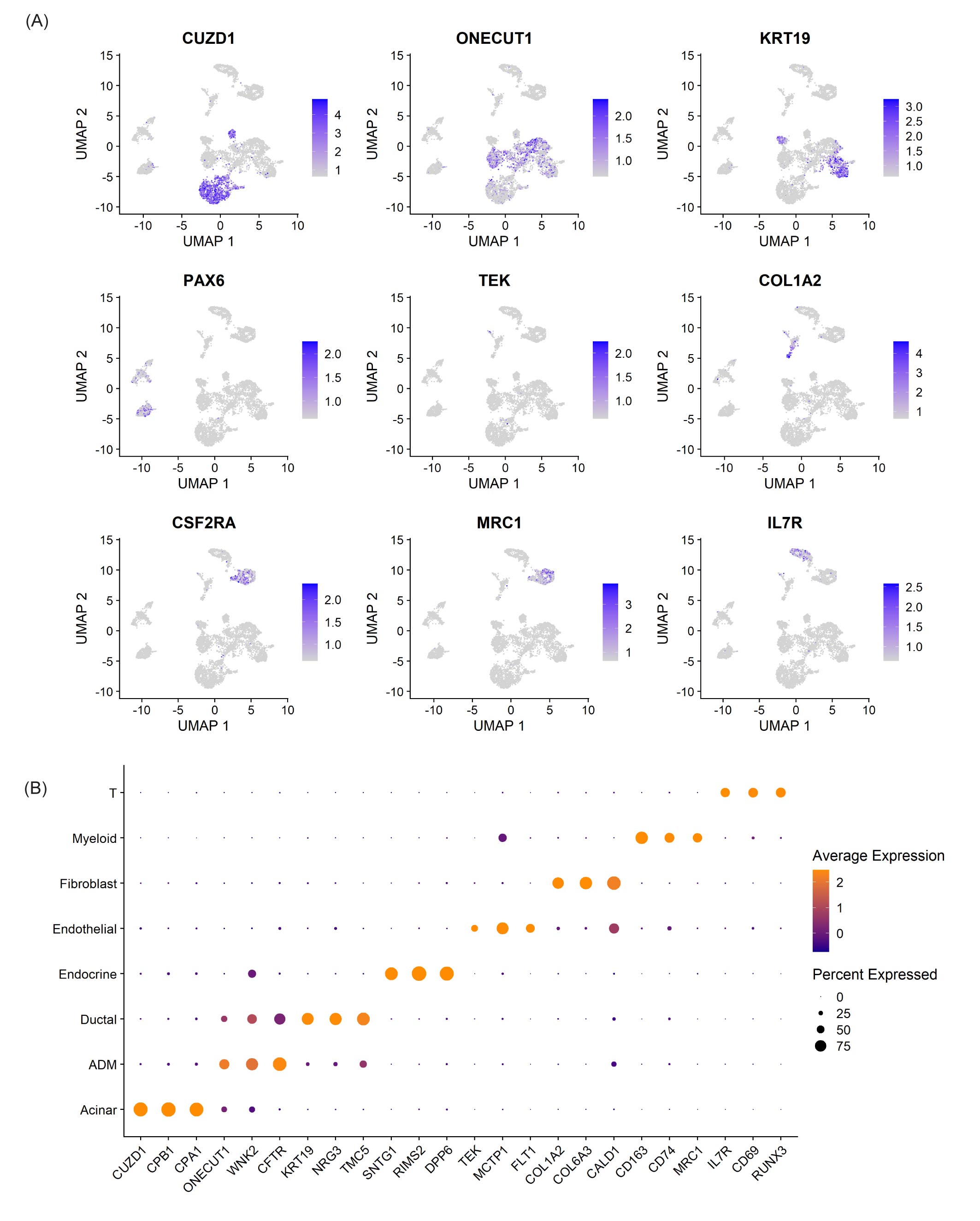


**Supplemental Fig. 4 |** **Marker genes selected for cell type annotation for the snMultiome dataset**. (A) Uniform manifold approximation and projection (UMAP) visualization of the expression of cell type specific marker genes supported in the literature. (B) Bubble plot showing average expression of selected genes that are differentially expressed for each cell types (including some marker genes) and the percentage of cells expressing the genes.


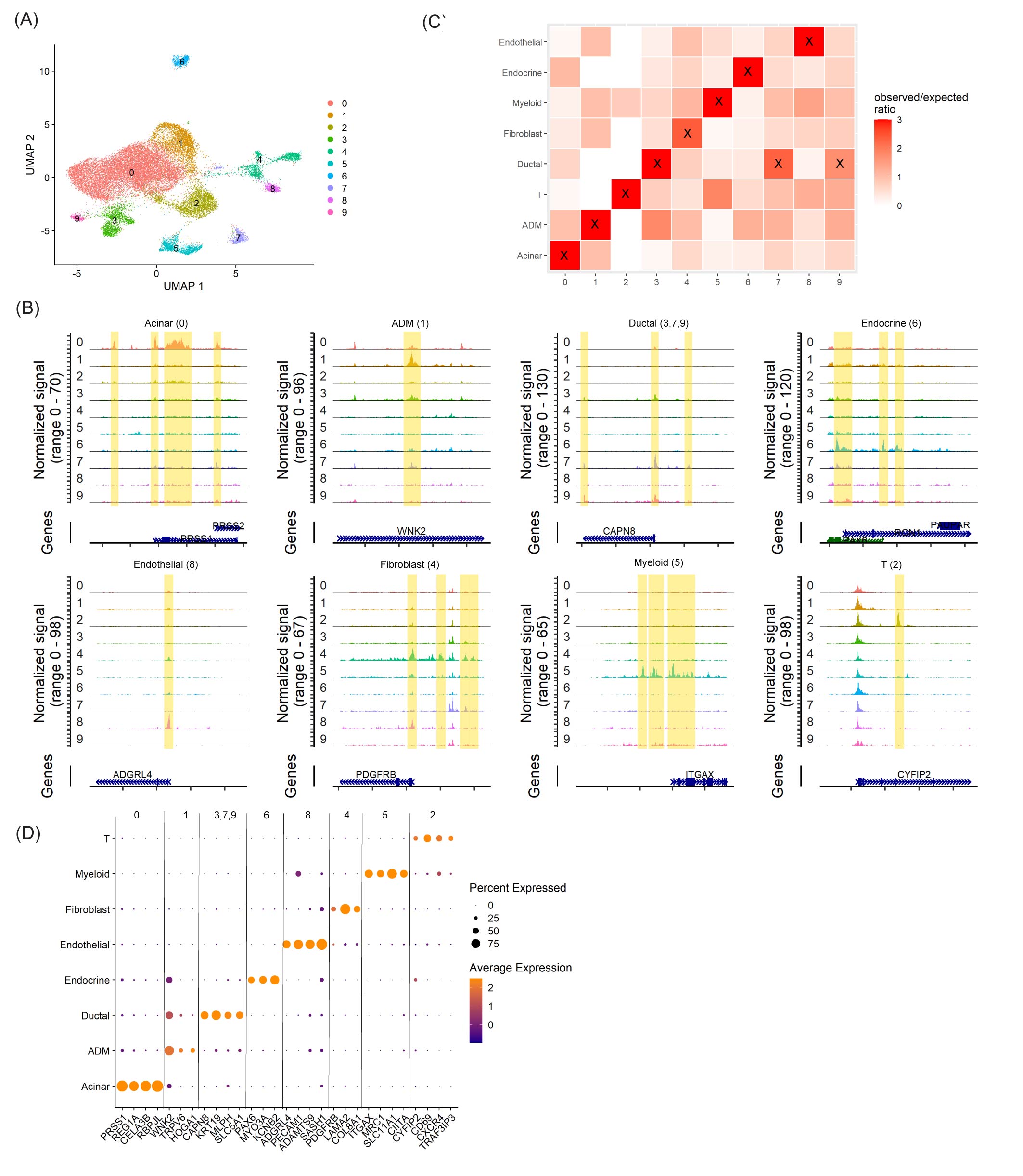


**Supplemental Fig. 5 |The cell type identification of snATAC-seq**. (A) UMAP visualization of the subclusters of single nucleus ATAC-seq for all benign and tumor samples. Each dot represents a single nucleus; colors indicate the clusters with numbered labels. (B) Genome browser tracks for representative subcluster specific chromatin accessible regions and their associated genes. Yellow shadowed are genomics regions showing differential chromatin openness in the snATAC-seq subcluster indicated in parenthesis at the top with cell type annotation. (C) Heatmap visualization of the fold enrichment (observed/expected) of marker genes predicted for each snATAC-seq subcluster (columns) when overlapping to the signature genes for each cell type annotated from snMultiome data (the RNA part; rows). For each snATAC-Seq subcluster, the cell type was transferred from the scMultiome data by the one showing the highest fold-enrichment (marked as X). (D) Bubble plot for the expression pattern of representative marker genes predicted for each scATAC-seq subcluster (marked on x-axis) within the cells from the snMultiome data according to cell types (y-axis). These genes are top signature genes identified from snATAC-seq data based on their cluster-specific chromatin accessibility occurred at promoters.

**
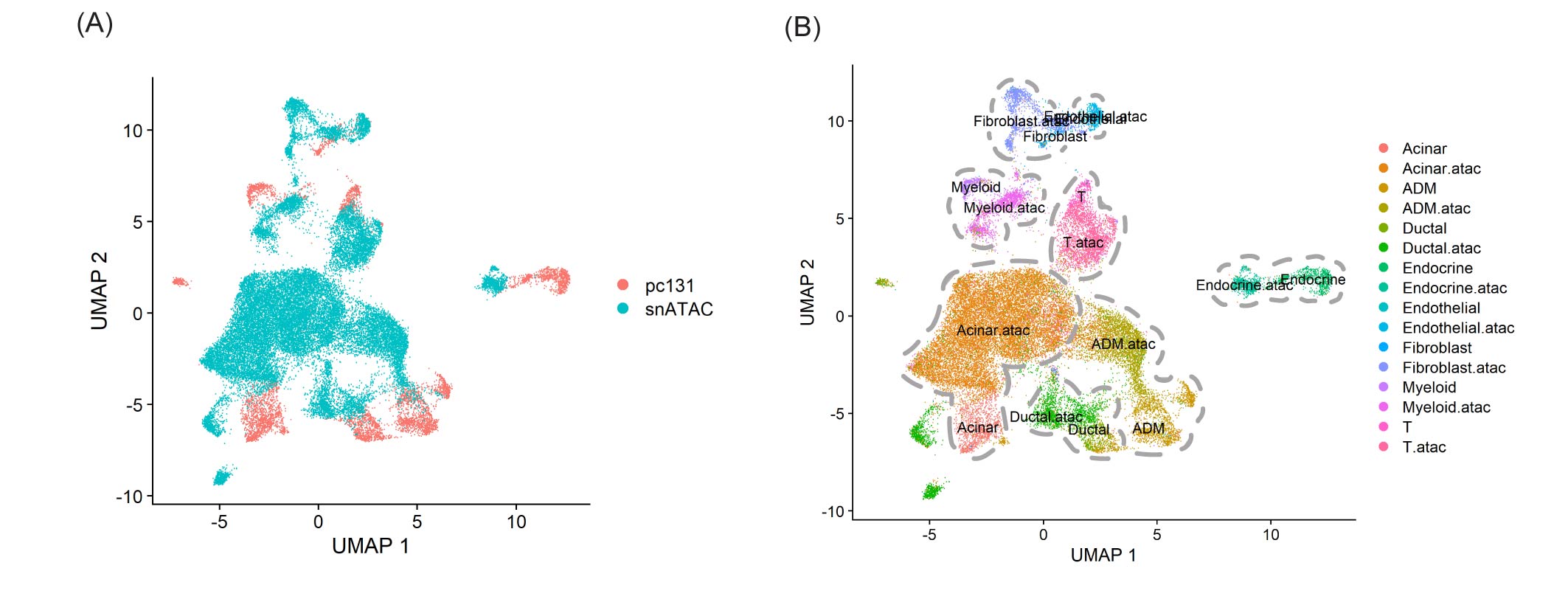
**

**Supplemental Fig. 6 |** **Cell type validation through snATAC-seq and snMultiome ATAC part integration.** (A) UMAP visualization of the snATAC-seq and snMultiome ATAC part integration. Colors indicate the source of dataset. Pc131represents the ATAC part of snMultiome dataset, and snATAC represents the four benign and four tumor snATAC-seq datasets. (B) UMAP visualization of the integration dataset. Colors indicate the cell type. The cell type name with the atac as the suffix represents cells from the snATAC-seq dataset and their annotations. No suffix indicates cells from the snMultiome data set and their annotations. Cell type proximity is indicated by gray circles as an example.


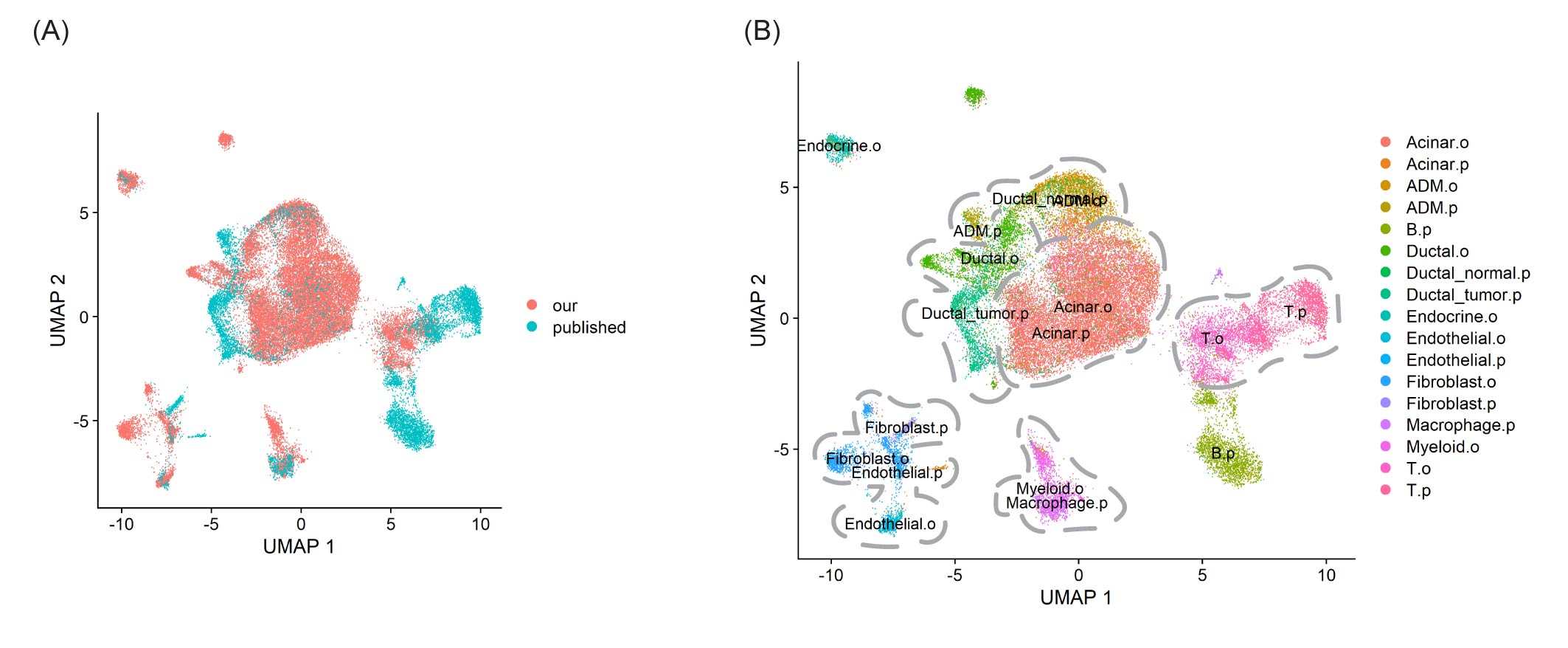


**Supplemental Fig. 7 | Cell type validation through snATAC-seq and published scATAC-seq integration.** (A) UMAP visualization of the snATAC-seq and published scATAC-seq integration. Colors indicate the source of dataset. “Our” means our snATAC-seq dataset and “published” means the published scATAC-seq datasets (GSE147726).  (B)  UMAP visualization of the integration dataset. The cell type name with the ‘o’ as suffix indicates cells from our snATAC-seq data set and their annotations, and suffix ‘p’ means cells from the published data set and their annotations. Manually circled are clusters with cell types shared between the two sources.


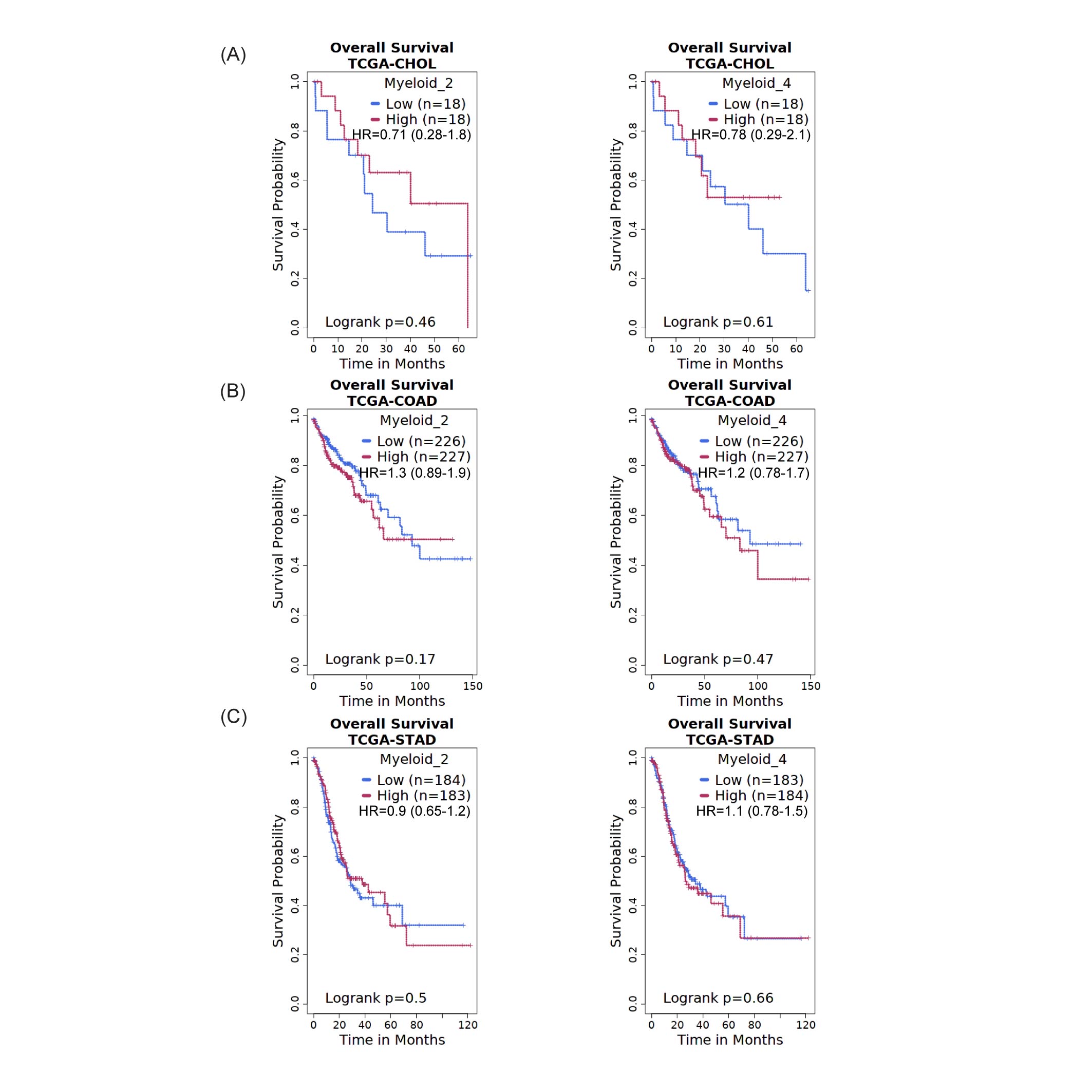


**Supplemental Fig. 8 | Kaplan-Meier survival curves for gastrointestinal malignancies.** (A-C) Kaplan-Meier analysis using signature genes for Myeloid subcluster 2 (dendritic cells) and Myeloid subcluster 4 (Tissue resident/epithelial-like macrophage) applied to patient cohorts of TCGA-CHOL (A), TCGA-COAD (B), TCGA-STAD (C).
