# Supplementary material for: Analysis of single nuclear chromatin accessibility reveals unique myeloid populations in human pancreatic ductal adenocarcinoma
Source: Clin Transl Med. 2024 Mar 1;14(3):e1595. doi: 10.1002/ctm2.1595 (PMC10905544; doi:10.1002/ctm2.1595)
Supplement: Supplementary file 7 — Supporting Information [file CTM2-14-e1595-s001.docx]

**Tables**

**Supplementary Table 1:**

The marker genes for each cell type, which were identified by the RNA part data of snMultiome. Columns include p-value (p_val), log2 fold chance (avg_log2FC), cluster identification (cluster), and gene name (gene).

**Supplementary Table 2:**

The differentially accessible regions for annotation of the 4 benign and 4 tumor snATAC-seq dataset. Sheet all_regions includes all open regions and sheet promoter_regions filters for differentially open promoter regions. Columns for chromosome (chr), start of opening (start) and end of opening (end), chromosomal region (region), p-value (p_val), log2 fold change (avg_log2FC), cluster number (cluster), type of opening (annotation), gene identification (geneID), transcript identification (transcriptID), distance to transcription start site (distanceTOTSS), gene symbol (SYMBOL), and gene name (GENENAME) are included. Sheet subcluster_promoter_genes provides a list of genes open separated by cluster.

**Supplementary Table 3:**

The differentially accessible regions and their annotation of the subclusters of myeloid subpopulation from the tumor snATAC-Seq dataset. Sheet all_regions includes all open regions and sheet promoter_regions filters for differentially open promoter regions. Columns for chromosome (chr), start of opening (start) and end of opening (end), chromosomal region (region), p-value (p_val), log2 fold change (avg_log2FC), cluster number (cluster), type of opening (annotation), gene identification (geneID), transcript identification (transcriptID), distance to transcription start site (distanceTOTSS), gene symbol (SYMBOL), and gene name (GENENAME) are included. Sheet subcluster_promoter_genes provide a list of the genes for subcluster 1-4, and genes (bold, red) used for survival analysis.

**Supplementary Table 4:**

The GO Enrichment for all the nuclei clusters from benign and tumor. Sheets are labeled by cluster. Columns for Description, PARENT_GO, LogP, Enrichment, Z-score, and Hits were included.

**Supplementary Table 5:**

The GO Enrichment for nuclei from myeloid subclusters. Sheets are labeled by cluster. Columns for Description, PARENT_GO, LogP, Enrichment, Z-score, and Hits were included.
